# VC@Scale: Scalable and high-performance variant calling on cluster environments

**DOI:** 10.1093/gigascience/giab057

**Published:** 2021-09-07

**Authors:** Tanveer Ahmad, Zaid Al Ars, H Peter Hofstee

**Affiliations:** Faculty of Electrical Engineering, Mathematics and Computer Science, Quantum & Computer Engineering Department, Mekelweg 4, 2628 CD Delft, Netherlands; Faculty of Electrical Engineering, Mathematics and Computer Science, Quantum & Computer Engineering Department, Mekelweg 4, 2628 CD Delft, Netherlands; Faculty of Electrical Engineering, Mathematics and Computer Science, Quantum & Computer Engineering Department, Mekelweg 4, 2628 CD Delft, Netherlands; IBM Austin, TX, USA

**Keywords:** whole-genome sequencing, Apache Spark, Apache Arrow, BWA-MEM, sorting, MarkDuplicate, DeepVariant

## Abstract

**Background:**

Recently many new deep learning–based variant-calling methods like DeepVariant have emerged as more accurate compared with conventional variant-calling algorithms such as GATK HaplotypeCaller, Sterlka2, and Freebayes albeit at higher computational costs. Therefore, there is a need for more scalable and higher performance workflows of these deep learning methods. Almost all existing cluster-scaled variant-calling workflows that use Apache Spark/Hadoop as big data frameworks loosely integrate existing single-node pre-processing and variant-calling applications. Using Apache Spark just for distributing/scheduling data among loosely coupled applications or using I/O-based storage for storing the output of intermediate applications does not exploit the full benefit of Apache Spark in-memory processing. To achieve this, we propose a native Spark-based workflow that uses Python and Apache Arrow to enable efficient transfer of data between different workflow stages. This benefits from the ease of programmability of Python and the high efficiency of Arrow’s columnar in-memory data transformations.

**Results:**

Here we present a scalable, parallel, and efficient implementation of next-generation sequencing data pre-processing and variant-calling workflows. Our design tightly integrates most pre-processing workflow stages, using Spark built-in functions to sort reads by coordinates and mark duplicates efficiently. Our approach outperforms state-of-the-art implementations by >2 times for the pre-processing stages, creating a scalable and high-performance solution for DeepVariant for both CPU-only and CPU + GPU clusters.

**Conclusions:**

We show the feasibility and easy scalability of our approach to achieve high performance and efficient resource utilization for variant-calling analysis on high-performance computing clusters using the standardized Apache Arrow data representations. All codes, scripts, and configurations used to run our implementations are publicly available and open sourced; see https://github.com/abs-tudelft/variant-calling-at-scale.

## Introduction

Immense improvements in next-generation sequencing (NGS) technologies enable large amounts of high-throughput and cost-effective raw genome datasets to be produced. On the one hand, this development paves the way to analyze more genomes with higher accuracy, but at the same time this creates the computational challenge of processing such a large amount of data in a timely fashion. The approximate raw data size of the human genome sequenced using NGS technologies is 300 Gb when sequenced with 30× coverage and can be >1 TB raw data with 300× sequencing coverage. The ongoing pace of development of these technologies promises even longer reads of up to 100 kb and with more coverage depth.

To process and prepare raw data for downstream analysis, many open-source and proprietary bioinformatics tools and workflows are available to run on single-node machines. But due to the continuous growth in genomics datasets, processing these data on a single node becomes inefficient and time consuming because of input/output (I/O) bottlenecks, limitations on the number of physical cores in a single CPU, and memory capacity constraints. To scale up these tools for distributed computing environments, both high-performance computing (HPC) programming models (using Message Passing Interface [MPI]) and big data frameworks (using Hadoop and Spark) have been explored in the past decade.

MPI implementations leverage the benefits of distributed memory architectures in inter-node communication. The workflow can exploit the maximum bare-metal performance of such multi-node clusters using shared memory MPI implementations. Previously, insufficient emphasis has been put on developing MPI-based cluster-scaled bioinformatics tools and workflows. The reason can be the lack of fault tolerance [1], redundant data replication, and the complexity of developing parallel algorithms in this approach. However, new fault tolerance models for MPI [2] can enable fault tolerance mechanisms for such applications and workflows. Similarly, the availability of 1-sided communication in new the MPI-3 RMA (Remote Memory Access) standard promises better performance gains in the applications while requiring no (or very little) inter-node data sharing and communication. Many tools in a variant-calling workflow exhibit such a property of not sharing data between the nodes and can run independently (with the exception of sorting).

Apache Hadoop** [3]** is a MapReduce framework used to process chunks of big datasets in parallel on large cluster nodes in a fault-tolerant and reliable manner. MapReduce usually splits the input data into smaller chunks, runs these chunks completely independently in map tasks, and sorts the output of these tasks, which is fed to a reduce task as input to generate the final output. MapReduce exclusively uses key-value pair input data to process, sort, and aggregate the output on the basis of keys. Hadoop Distributed File System (HDFS) is commonly used to store the input and output data on local compute nodes or on network storage nodes. Some early variant-calling workflows such as Halvade [4] use this approach to exploit computing cluster resources by running multiple legacy application instances (loosely integrated in the Apache Hadoop Framework) in parallel on chunked input data.

Apache Spark** [5]** is a unified analytics engine to process big data in a distributed computing environment, with built-in modules for streaming data, distributed machine learning, SQL functions, and graph processing. Spark also provides high-level APIs for Java, Scala, Python, and R languages. In Spark, resilient distributed datasets (RDDs) are the core components that are distributed across the nodes of a cluster to be operated on in parallel. RDDs can be cached/persisted in-memory across nodes to store intermediate results for iterative processing. Spark commonly uses HDFS to read/write data but also supports other storage systems such as Network File System (NFS), HBase, and Amazon’s S3. Many variant-calling workflows and tools have been developed over the past decade since its first release, including SparkGA2 [6], ADAM [7], SparkBWA [8], BWASpark [9], PipeBWA [10], and others.

In this article, we propose and implement a new framework that combines the advantage of easy programmability of Apache Spark and the high efficiency of MPI. The resulting framework integrates Apache Spark NGS data pre-processing with the Apache Arrow in-memory data format. Our framework tightly integrates pre-processing (read sorting and duplicate removal) applications in Python using distributed Dataframes (DF)-based sorting and vectorization. This is the first ever such implementation for genomics data to exploit the benefits of Apache Arrow in-memory data format in Apache Spark. The key contributions of our approach are as follows:

- The first scalable approach for DNA data pre-processing that uses Apache Arrow for efficiently utilizing compute resources while preserving easy programmability
- Improved performance of up to 2 times compared with state-of-the-art scalability approaches
- Integration with DeepVariant to create the first scalable open-source DeepVariant workflow on Apache Spark

This article is organized as follows. In Section “Background and Related Work,” we discuss single-node and cluster-scaled pre-processing and variant-calling workflows, followed by the Methods section, which presents the in-depth details of the new Apache Arrow–based data format for NGS data. In Section “Design and Implementation,” the internal design flow and implementation details of our new efficient workflow are discussed. Furthermore, Section “Results and Evaluation” describes the results of our implementation using different node configurations with different sequencing coverage/depth datasets to show the scalability and the performance comparisons with state-of-the-art methods. In the Discussion section, more detailed insights on performance, scalability, resource utilization, and memory consumption are given. Finally, the Conclusion provides some concluding remarks and possible future directions.

## Background and Related Work

In this section, first we introduce and discuss some tools used to pre-process NGS data followed by a discussion of some widely used cluster-scale variant-calling workflows.

### Pre-processing NGS data

Pre-processing of NGS data requires a number of steps: (i) alignment of raw FASTQ data against a reference genome, (ii) chromosome-based coordinate sorting, and (iii) PCR duplicate removal (optional, only required if data are not PCR-free or in some datasets for better accuracy). These steps are common to almost every variant-calling workflow. There are many publicly available tools that can pre-process NGS data efficiently on single-node machines. Bowtie2 [11] and BWA-MEM [12] tools are widely used for short-read sequence alignments. SAMtools [13], Picard [14], Sambamba [15], and samblaster [16] are some of the most famous and widely used tools for the purpose of indexing, sorting, and duplicate removal in SAM/BAM/CRAM files.

### Variant calling

**Figure 1:**
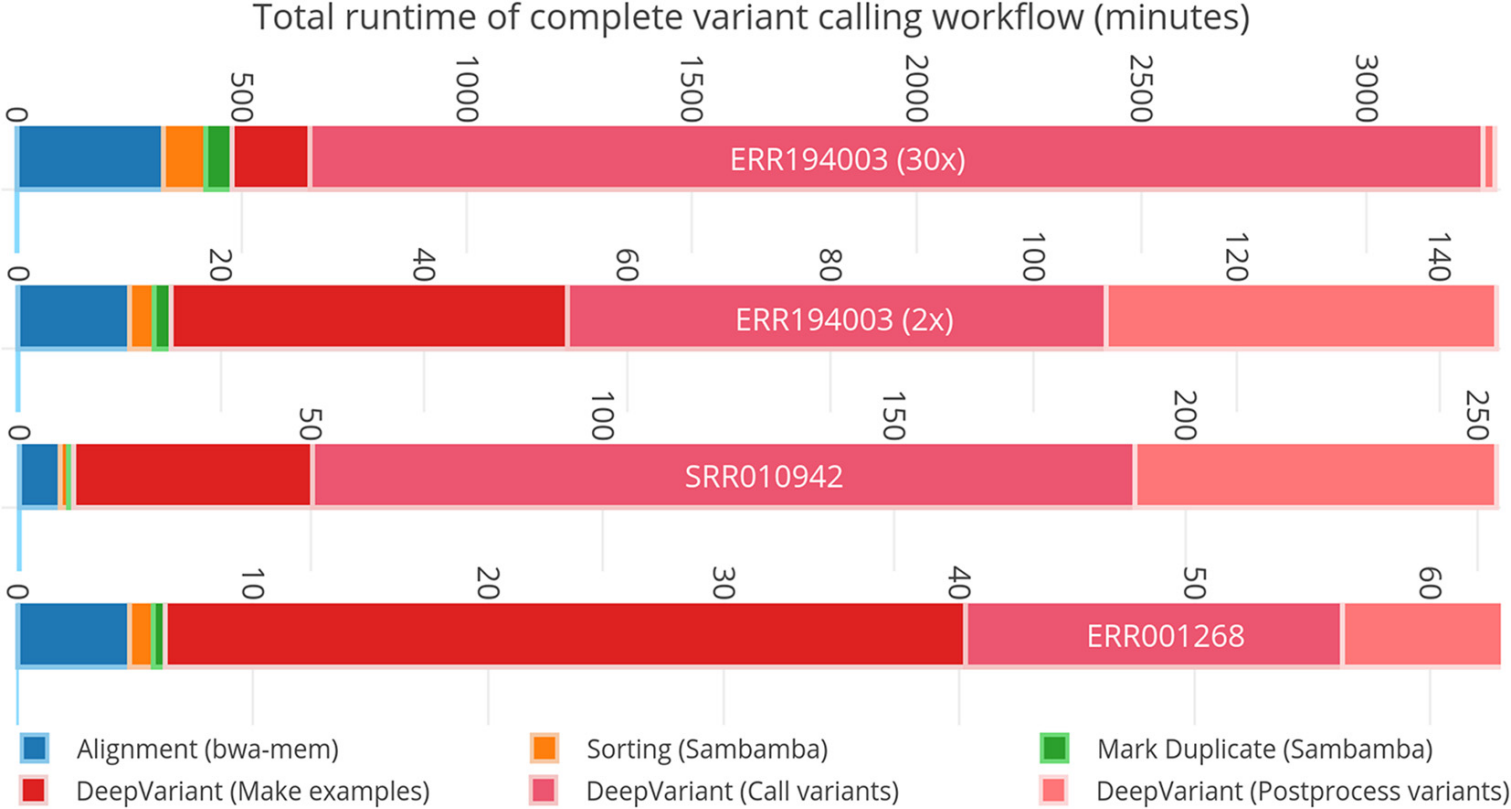
Single-node total runtimes for complete variant-calling workflow using DeepVariant for different datasets.

Variant calling reveals deep insights into nucleotide-level organismal differences in some specific traits among populations from an individual's genome sequence data. It discerns genetic variations in 3 categories: single-nucleotide polymorphisms (SNPs), insertions and deletions (indels), and/or structural variants (SVs; may also include copy number variations [CNVs], duplication, translocation, and so forth). The GATK HaplotypeCaller is a widely used variant caller for detecting germline variations. DeepVariant [17] is being considered a more accurate germline variant caller for both short and long reads. Such tools as VarScan [18], VarDict [19], and MuTect2 [20] are used for somatic variant-calling analysis. NeuSomatic [21,22] is a deep convolutional neural network–based somatic variant caller that runs in both stand-alone and ensemble modes (MuTect2, MuSE, Strelka2, SomaticSniper, VarDict, and VarScan2) for accurate somatic variant detection. Octopus [23], FreeBayes [24], Strelka2 [25], SNVer [26], and LoFreq [27] are also used for both germline and somatic variant-calling analysis. The DeepVariant variant caller–based workflow outperforms all other methods in both PrecisionFDA (pFDA) Challenges v1 [28] (highest SNP performance) and v2 [29] (all benchmark regions for Pacific Biosciences and multiple, difficult-to-map regions for Oxford Nanopore Technology). DeepVariant does not require any additional pre-processing steps such as base quality recalibration. Therefore we selected this variant caller to integrate with our pre-processing workflow. As shown in Fig. 1, we run the fastest pre-processing tools with DeepVariant on a single machine with different datasets to get an idea of individual tool runtime in the workflow.

### Cluster-scaled workflows

There are many cluster-scaled multi-node implementations available for alignment using both HPC languages such as MPI/Unified Parallel C (UPC), as well as big data frameworks such as Hadoop MapReduce and Apache Spark. pBWA [30] and mpiBLAST [31] use MPI, and CUSHAW3 [32] uses UPC++. Similarly ADAM’s Cannoli [7], SparkBWA [8], and PipeMEM [10] are a few Apache Spark–based BWA implementations that use BWA as loosely integrated underneath these implementations while GATK BWASpark modifies the original BWA to exploit the Spark scheduling and shuffling functionality to run BWA instances in parallel on clusters.

ADAM, Halvade, and SparkGA2 are a few implementations that also handle whole variant-calling workflows based on GATK best practices including alignment, sorting, duplicate removal, and base quality score recalibration.

ADAM, Halvade, and SparkGA2 use the built-in Scala API in Spark for sorting the aligned reads. Because the Picard MarkDuplicate algorithm is considered the standard for paired-end reads for duplicate removal, SparkGA2 and Halvade use this Picard MarkDuplicate tool in Spark for distributed processing on clusters, while ADAM has implemented their own duplicate removal algorithm in Scala, which is nearly identical to the Picard MarkDuplicate algorithm. A more detailed comparison of these workflows for each individual pre-processing stage output storage strategy is given in Table 1.

**Table 1:** A comparison of NGS data pre-processing workflows with their output storage approaches for each stage

Framework	Alignment (output)	Sorting (output)	Duplicate removal (output)
Halvade	*.SAM in disk	In-memory (elPrep)	In-memory (elPrep)
SparkGA2	*.fq.gz in disk	*.BAM in disk	*.BAM in disk
ADAM	ADAM Parquet in disk	ADAM Parquet in memory	ADAM Parquet in memory
VC@Scale (this work)	In memory (Apache Arrow RecordBatches)	In memory (PySpark DFs)	In memory (PySpark DFs → *.BAM)

### Apache Arrow in Apache Spark

**Figure 2:**
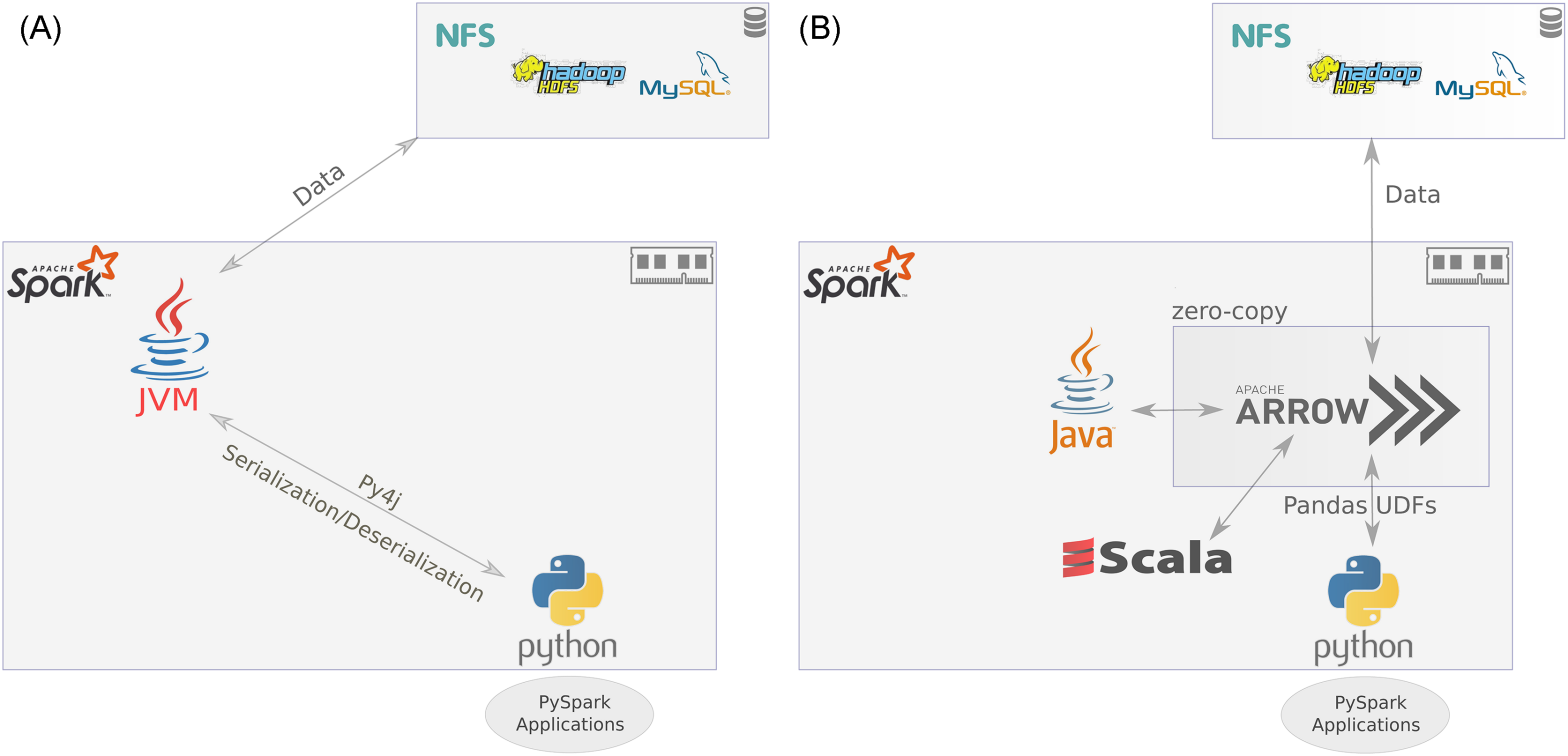
A. Python programs in Spark require inefficient data serialization/deserialization between Python and JVM processes (using the Py4j library). B. Efficient data communication between frameworks/languages using Apache Arrow unified in-memory columnar data format with zero-copy overhead and different languages APIs/interfaces availability in Spark cluster.

**Apache Arrow [33]** is an in-memory standard columnar data format. Apache Arrow also provides API interfaces and functions to process datasets in Go, C, C++, C#, Java, JavaScript, R, Rust, MATLAB, Ruby, and Python languages. Owing to the columnar data storage, efficient vectorized data analytics operations and better cache locality can be exploited. This in-memory format also supports zero-copy reads for large datasets in inter-process communication without serialization/deserialization overheads. Figure 2 shows how a common Apache Arrow–based data format is being used in Apache Spark with different language interfaces.

#### Apache Spark leveraging Apache Arrow

In this article, we use Python as the language to implement our workflow owing to its high level of abstraction and ease of implementation. It also has a stable API to Apache Arrow [34] used in Apache Spark to efficiently transfer data between JVM and Python processes.

#### Pandas user-defined functions

The Python computation model in PySpark on user-defined functions (UDFs) is scalar; i.e., during UDF evaluation, the JVM executor process sends row data to PySpark workers, which invoke UDFs on a row-by-row basis and send the results back to the executor process. However, the current Spark/PySpark release uses immutable Arrow RecordBatches (RBs) data instead of Spark built-in row-based data. This enables vectorized UDF evaluation on these RBs using Pandas Dataframes, which in turn gives a huge performance improvement. Owing to vectorized UDF operations, the reduced number of system calls enables faster I/Os.

Because traditionally Apache Spark uses a row-based memory layout, using Arrow RBs requires converting Spark row-based data to Arrow RecordBatch and vice versa to apply vectorized UDF operations in Pandas Dataframes. Some other operations (e.g., grouped data in Pandas Dataframes on UDFs, and converting Spark Dataframes to/from Pandas Dataframes) are also becoming more efficient using Arrow underneath, which is discussed in more details in the Methods section.

#### Pandas function APIs

Python native functions can be applied on PySpark Dataframes, which input/output Pandas instances. Grouped map, map, and cogrouped map are a few Pandas API functions to apply on PySpark Dataframes. These functions use Arrow to transfer data and Pandas to work on those data. These functions share the same characteristics as those of Pandas UDFs.

#### UDF performance with or without Arrow

**Figure 3:**
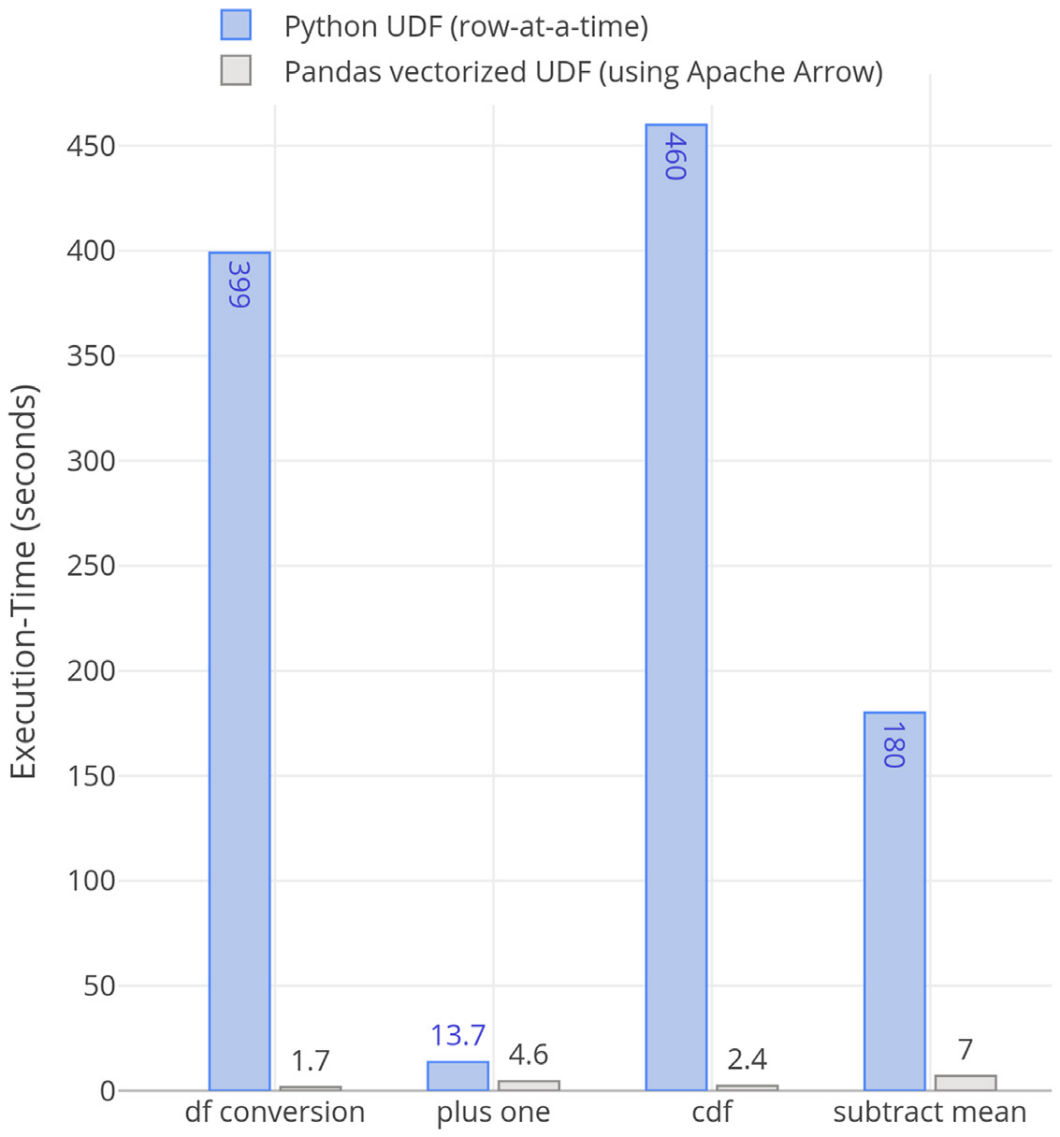
Performance comparison of Pandas dataframe to PySpark dataframe conversion using Arrow and without Arrow and Python UDF (row-at-a-time) and Pandas vectorized UDF (using Apache Arrow) operations: plus one, cdf, and subtract mean.

The Spark Python API supports UDFs that operate 1 row at a time, resulting in a large serialization and invocation overhead. Apache Arrow–based unified memory format brings the benefits of high-performance and low-overhead dataframes conversion (PySpark ↔ Pandas) and vectorized Pandas UDFs operations in Python native environments. Because Spark inherently operates on row-based memory layouts and Arrow data format is columnar, this requires row-column conversions (Spark row ↔ Arrow RecordBatch) overhead when doing these operations. In Fig. 3, we show the performance comparison of (i) converting a Pandas dataframe to PySpark dataframe with Arrow and without Arrow, (ii) Python UDF (row-at-a-time) and Pandas vectorized UDF (using Apache Arrow) for plus 1, (iii) cumulative probability distribution function (cdf), and (iv) subtract mean examples [35].

## Methods

In this section, we discuss the details of architectural approaches that we have adopted in this work for processing the variant-calling workflow.

### Overview

The benefits of using distributed big data frameworks to process genomics data are 4-fold: they provide easy and flexible deployment, efficient cluster scalability, and fault tolerance, as well as cheaper costs on public clouds and private HPC clusters. Traditionally, these frameworks use distributed file systems like HDFS or NFS for storage. The intermediate processing stages place data in-memory on-demand if enough memory is available in the form of RDDs. RDDs generally store data in their internal row format while Apache Arrow provides an efficient columnar data format to create distributed RDDs of Arrow RecordBatches object types.

To validate the scalability and performance advantage of our Apache Arrow–based in-memory data placement, shuffling, conversion, and computation techniques in Apache Spark using PySpark, we present the design methods for a full variant-calling workflow. We have also developed high-performance and scalable but very simple, portable, and stand-alone methods for BWA-MEM and DeepVariant scalability on HPC clusters using traditional I/O-based storage.

### Variant-calling workflow

In this subsection, we describe the various stages of the variant-calling workflow that we designed, as shown in Fig. 5. We start with the implementation of the pre-processing stages (alignment, sorting, and duplicate removal) using Apache Arrow in-memory data format for temporary data storage in Plasma Stores, shuffling/conversion of data, and transformations/computations on these data. The resultant data from these pre-processing stages is saved in BAM format. Each BAM file contains the reads of a particular chromosome and a specific region inside a chromosome. Variant caller (DeepVariant) instances process these BAM files on worker nodes and produce VCF files, which are merged to produce a final VCF file.

### FASTQ chunk streaming

We use the SeqKit [36] to create the FASTQ input chunks in parallel with BWA-MEM for input paired-end NGS data as shown in Step 1 of Fig. 5. SeqKit is an efficient multi-threaded utility, through which we provide these FASTQ data to BWA-MEM instances in streaming fashion, without the need to create FASTQ chunks separately. The number of created FASTQ chunks can be configured in the SeqKit command option, depending on the number of nodes available in the Spark cluster.

### Arrow integration in BWA-MEM

BWA-MEM is the most popular alignment tool in the bioinformatics community owing to its efficient and accurate alignment algorithm for short reads. In our implementation, each Spark cluster worker node runs 1 BWA-MEM instance as shown in Step 2 of Fig. 5. We have modified BWA-MEM to output in-memory key-value pair SAM data instead of creating tab-delimited SAM files.

#### Key-value pairs

Key-value pair-based data have proven efficient sorting performance as compared with text/columnar data structures. For every read, after creating its respective SAM fields we convert the whole read SAM data into a key-value pair <POS:SAM> and with RNAME, an extra piece of information in the structure to store it in a designated immutable Arrow RecordBatch. Each RecordBatch is a combination of a schema, which specifies the types of data fields, and the data item itself. In our case, the POS field is integer (Int) type while the SAM and RNAME fields are String type.

#### Static load balancing

**Figure 4:**
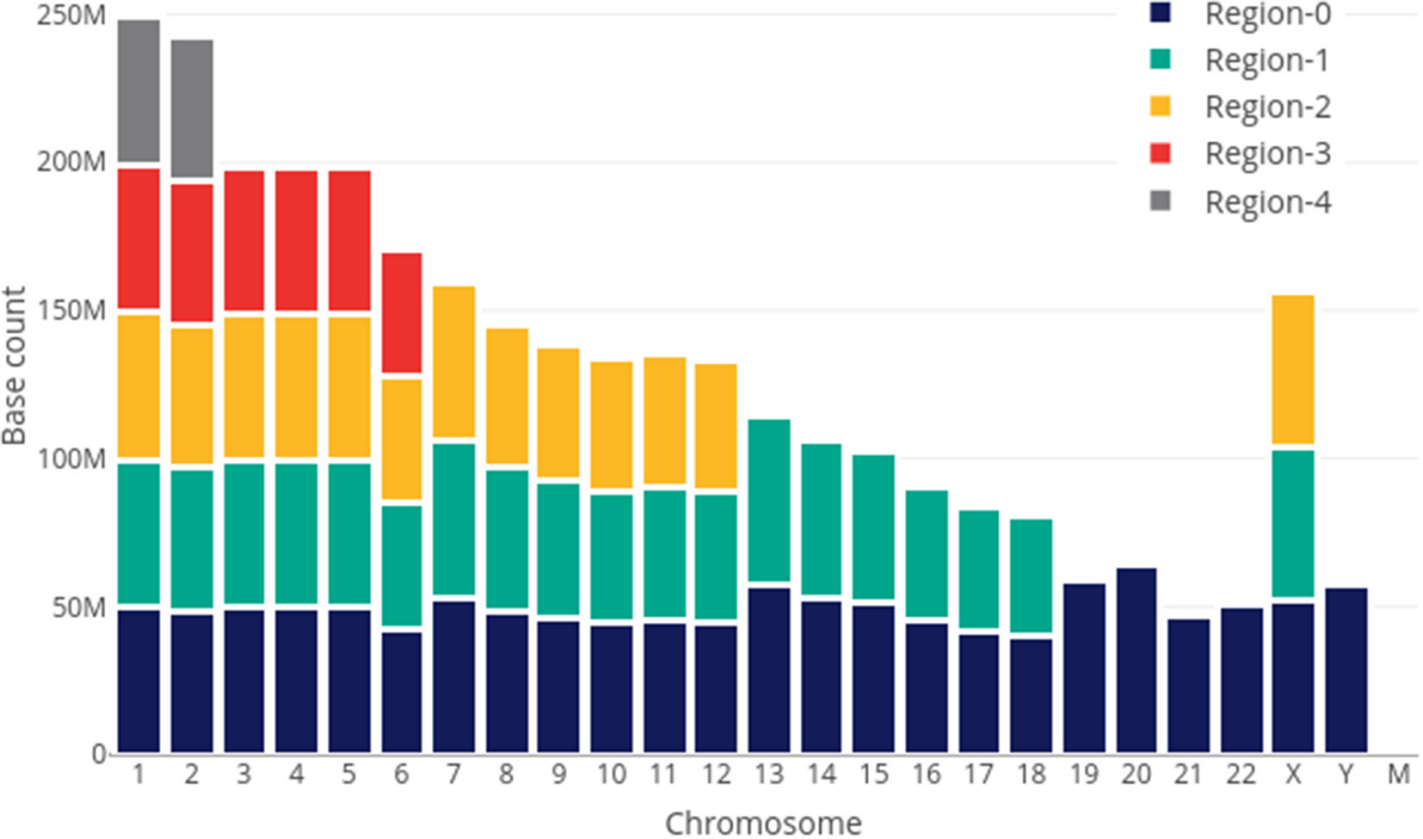
Static load balancing technique adopted in this work for BWA-MEM output, which divides chromosome-based regions to join and process them in parallel for all further workflow stages.

Owing to the size differences in the chromosomes of the human genome, we created chromosome regions for efficient scalability in BWA-MEM, and the same trend is followed in subsequent pre-processing stages as well. The number of regions is different for each chromosome; reads are stored corresponding to their respective regions as shown in Fig. 4. Each region in each chromosome is on average equal to 40–50 million base pairs.

#### Plasma Object Store

**Figure 5:**
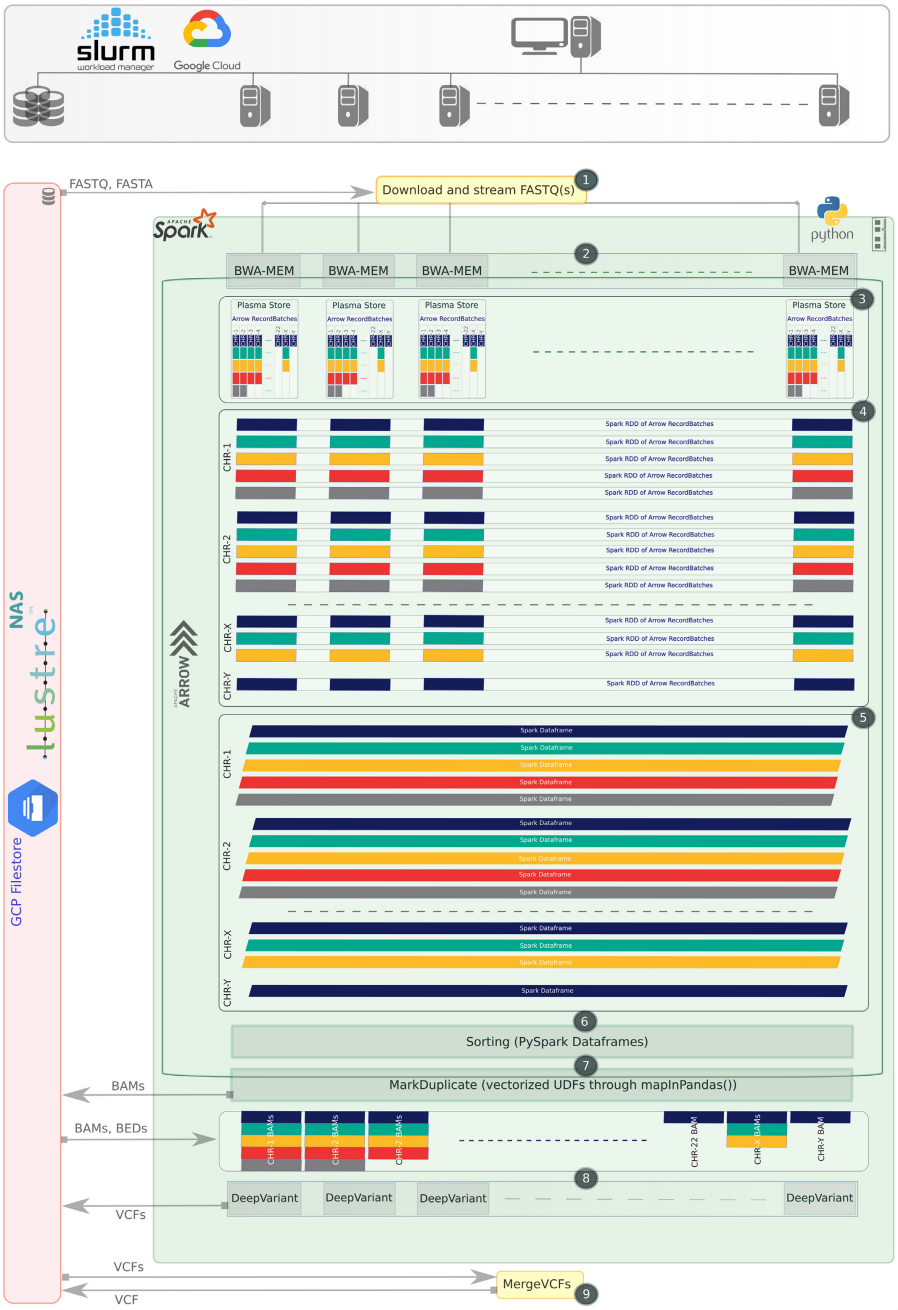
Complete design flow of the variant-calling workflow implementation in VC@Scale. This design encompasses Slurm Spark/GCP DataProc cluster, Lustre/GCP Filestore as file system, Apache Arrow as in-memory data format for pre-processing, and DeepVariant as variant caller.

The Plasma Object Store is an inter-process communication component of Apache Arrow that handles shared memory pools across different heterogeneous systems [37]. To perform inter-process communication, processes can create Plasma objects inside the shared memory pool that are typically data buffers underlying an Arrow RecordBatch. We cannot use more than half of overall system memory for these Plasma Stores. Through the shared memory pool, Plasma enables zero-copy data sharing between the processes. The output SAM data from BWA-MEM instances on each node are stored in key-value pairs in respective chromosomal regions using the Arrow in-memory format as shown in Step 3 of Fig. 5.

#### flatMap() on BWA-MEM

We apply the PySpark flatMap() function on BWA-MEM instances, which use an already SparkContext parallelized/distributed collection of input FASTQ chunks described in Section “FASTQ chunk streaming.” All the BWA-MEM instances create Arrow RecordBatches of regions of individual chromosomes on their own respective nodes. These Batches are temporarily placed in Plasma Object Stores on each node.

#### RDDs of Arrow RecordBatches

As soon as the alignment process on Apache Spark worker nodes finishes, we create distributed RDDs of these Arrow RecordBatch objects available across all the Spark worker nodes as shown in Step 4 of Fig. 5. Each RDD occupies the RecordBatches of a particular chromosome (with its specific region) distributed among all the worker nodes. Arrow RecordBatches are filtered out in this step and cached into the Spark context of the master node.

#### RDDs to Dataframe

These RecordBatches in RDDs are serialized and a PySpark schema is generated through corresponding Arrow schema enclosed in these RecordBatches. Python object to Java object conversion on RDDs is then applied as shown in Step 5 of Fig. 5. Finally, these resultant RDDs are converted to Spark Dataframe through Scala PythonSQLUtils() methods. At this point, we have distributed Spark Dataframes of specific regions of each chromosome. We process these specific chromosome regions independently and in parallel in the next sorting and duplicate removal stages.

### Sorting

All the Spark Dataframes containing specific chromosome regions are sorted (Step 6 in Fig. 5) by coordinates through df[n].orderBy('beginPos', ascending=True) function. This function is very fast and efficient in sorting huge distributed Dataframes. All the Dataframes are sorted in parallel using the Python multiprocessing library Pool method.

### Duplicate removal

Duplicate removal algorithms in this implementation were written from scratch in Python for both single and paired-end reads. These algorithms are developed using Pandas UDFs to apply on PySpark Dataframes, which can use the Pandas function APIs (df[n].groupby().applyInPandas()) to leverage the benefits of Arrow for data transfer/conversion and transformations (Step 7 in Fig. 5). For paired-end reads, a Picard MarkDuplicate compatible algorithm has been developed. The accuracy of this algorithm is validated using different datasets, so that they can be used as a cluster-scalable replacement for the existing Picard MarkDuplicate algorithm.

### DeepVariant integration

DeepVariant is considered an accurate variant caller for detection of both SNPs and indel variants in germline datasets. Published results show that DeeVariant performs best for most PrecisionFDA Truth Challenge datasets [38]. We have observed that on a single node, DeepVariant scales very well up to 6–12 threads. Therefore we have enabled running multiple DeepVariant instances on each Spark worker node using the PySpark flatMap() function (Step 8 in Fig. 5). Each DeepVariant instance takes input BAM (and BED as well in case of whole-exome sequencing data) and reference FASTA from the I/O-based NFS and produces individual VCF/gVCF files.

### VCFs merge

Finally, the individual VCFs created through DeepVariant instances are merged (Step 9 Fig. 5) through Samtools to produce a final complete VCF file(s) for further downstream analysis.

### Stand-alone implementations

In addition to implementing the complete workflow, we can also use BWA-MEM and DeepVariant as scalable stand-alone implementations capable of scaling almost linearly on HPC clusters depending on the input data size and number of nodes available.

#### BWA-MEM

Almost all BWA-MEM cluster-scaled implementations (SparkBWA [8], BWASpark [9], PipeMEM [10], ADAM [7], and SparkGA2 [6]) run multiple BWA-MEM instances on each Spark worker node as Spark tasks, which degrades the underlying efficient single-node multi-threaded scalability of this tool. Instead we use 1 BWA-MEM instance on each Spark worker node, storing output SAM files on storage and merging these SAM files to generate a single output SAM file.

#### DeepVariant

We use Samtools to generate different BAM files representing chromosome regions from a single BAM file in accordance with our human chromosome region–based approach as discussed in Section “Static load balancing.” Similarly, we have divided the reference FASTA into individual chromosome-based FASTA files using faSplit [39]. The VCF/gVCF output files of these instances can be merged through Mergevcf or Samtools.

## Results and Evaluation

In this section, first we briefly describe the datasets and HPC infrastructure used in the evaluation of our techniques. In addition, we compare our results with other state-of-the-art frameworks for both pre-processing and variant-calling stages, followed by a detailed analysis and comparison of scalability, performance, and speed-ups with these frameworks.

### Datasets

We use multiple whole-genome sequencing (WGS) datasets with varying coverage depth to analyze the maximum possible scalability and performance of our methods. The first dataset is sample ERR001268 from the 1000 Genomes Project (Phase 3) Illumina HiSeq-generated WGS paired-end read data of NA12878 [40]. In addition, we used Illumina HiSeq 2000 paired-end NA12878 cell line data sequencing sample ERR194147 [41] with sequencing coverage of 30×. We also used 300× sequencing coverage WGS data from Genome in a Bottle (GIAB) aligned with novoalign for the Illumina HiSeq 300× reads for NA12878 [42] to analyze the scalability of DeepVariant. Human Genome Reference, Build 37 (GRCh37/hg19) [43], is used as a reference genome.

### Evaluation HPC cluster

All experiments and comparisons were performed on the SurfSara Cartesius [44] HPC cluster (part of the Dutch national supercomputing infrastructure). Each CPU-only node is equipped with a dual socket Intel Xeon Processor (E5-2695 v2 or E5-2690 v3) running at 2.4/2.6 GHz. Each processor has 12 physical cores with support of 24 hyper-threading jobs. Similarly, each CPU + GPU node is equipped with a dual socket Intel Xeon Processor (E5-2450 v2) running at 2.5 GHz and 2× NVIDIA Tesla K40m GPGPUs. Each processor has 8 physical cores with support of 16 hyper-threading jobs. A total of 64 GB (E5-2695 v2/E5-2690 v3) and 96 GB (E5-2450 v2) of DDR4 DRAM with a maximum of 59.7 GB/s bandwidth is available for the whole system. A local storage of 1 TB and the same amount of network attached storage is available on the system. All nodes are connected through Mellanox ConnectX-3 or Connect-IB InfiniBand adapter.

Lustre [45] distributed and parallel file system is attached to our evaluation HPC cluster. Lustre file system has performance similar to that of HDFS/YARN-based Hadoop cluster for shuffle-heavy workloads in Apache Spark.

Red Hat Enterprise Linux operating system is installed on all nodes. The Apache Spark cluster is created in deploy-mode “client" thorough Slurm [46] Workload Manager and all workflows are executed through bash scripts.

We also used a Google GCP DataProc cluster and Google cloud Filestore, a network attached storage (NAS) to reproduce and run this approach on public cloud environments. All the required applications are installed on Dataproc custom image, which is based on the DataProc 2.0.1-ubuntu18 operating system. A detailed description and quick start guide to run all methods in this approach are given on the project GitHub page.

### Pre-processing (BWA, sorting, duplicate removal)

**Figure 6:**
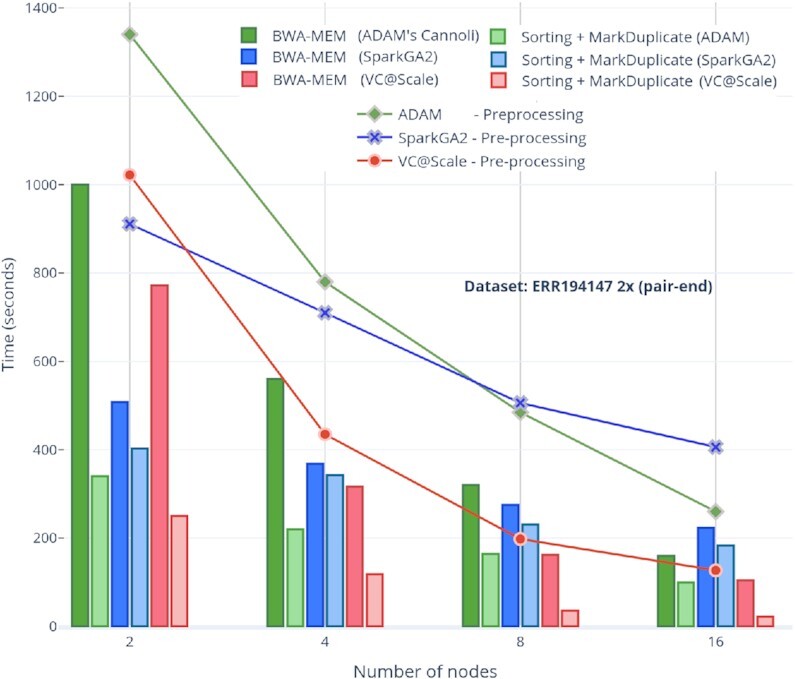
VC@Scale, SparkGA2, and ADAM comparisons of scalability for pre-processing stages using different number of nodes for ERR194147 (2×) dataset.

**Figure 7:**
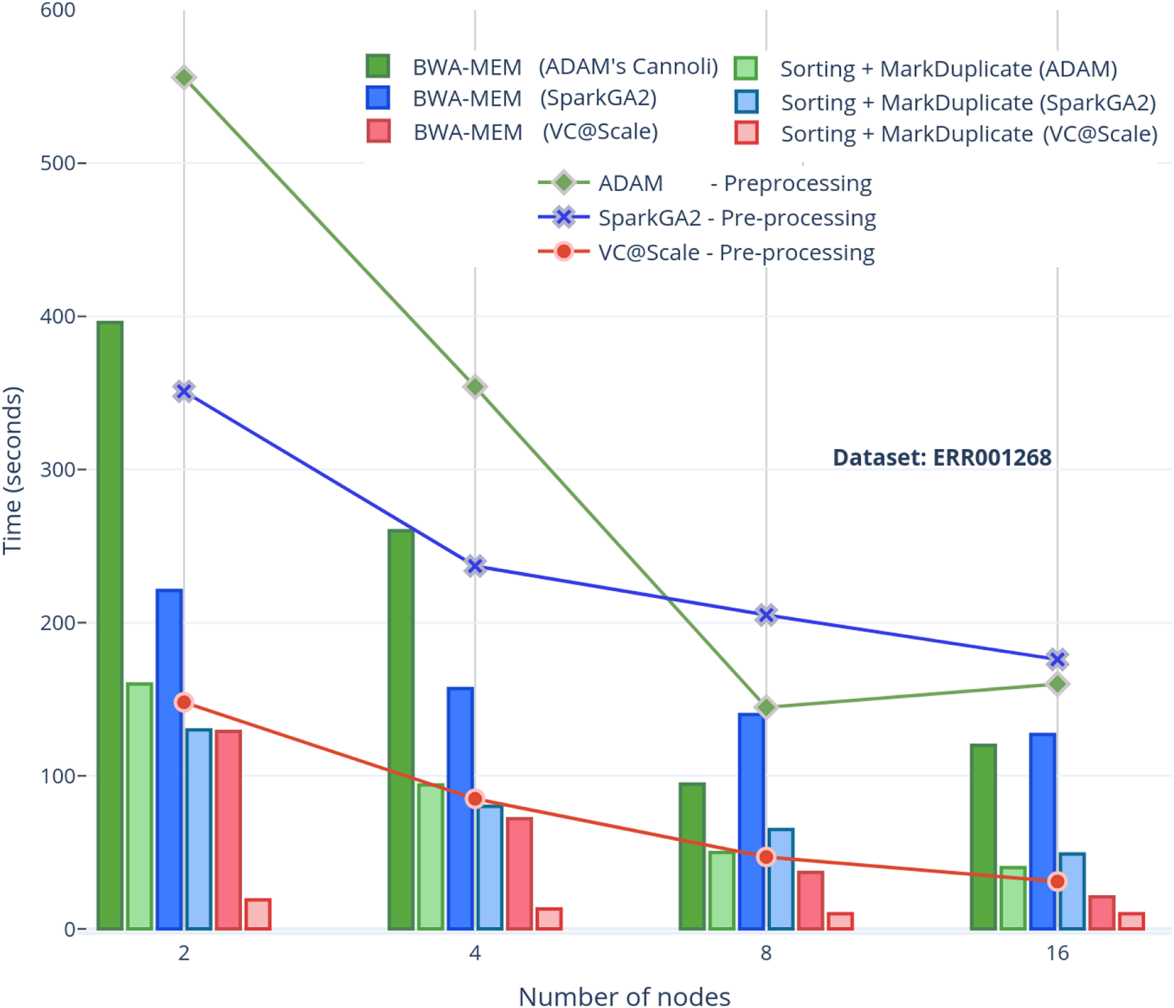
VC@Scale, SparkGA2, and ADAM comparisons of scalability for pre-processing stages using different number of nodes for ERR001268 dataset.

Our approach performs pre-processing in a more tightly coupled fashion (i.e., using native PySpark functions) as compared with alternative solutions such as SparkGA2, which stores the output of each of the pre-processing stages to storage and loads it again for subsequent stages. We have tested the scalability and performance of our architectural choices with that of SparkGA2 and ADAM for different cluster sizes; 2, 4, 8, and 16 nodes have been used in almost all comparisons. Storing BWA-MEM output to in-memory key-value pairs using the Arrow format involves almost zero cost overhead for loading data to the next sorting stage. The only data transformation that happens between the alignment and sorting stages is the conversion of RDDs containing Arrow RecordBatch objects to PySpark Dataframes. This transformation is handled through the Apache Arrow APIs internally. A similar key-value pair transformation of sorted Dataframes to SAM values occurs before the MarkDuplicate stage. Compared to SparkGA2 and ADAM pre-processing results, >2 times speed-up is achieved for all cluster sizes and for both ERR001268 and ERR194147 (2×) datasets for SparkGA2 while 2–4 times speed-up is achieved as compared to ADAM workflow pre-processing, as shown Figs   6 and 7, respectively.

### Variant calling (DeepVariant)

**Figure 8:**
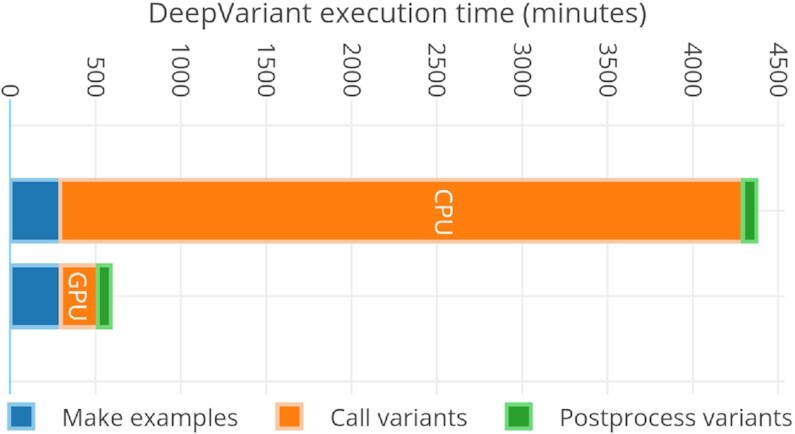
Single-node CPU-only and GPU accelerated DeepVariant for ERR194147 (30×) dataset.

**Figure 9:**
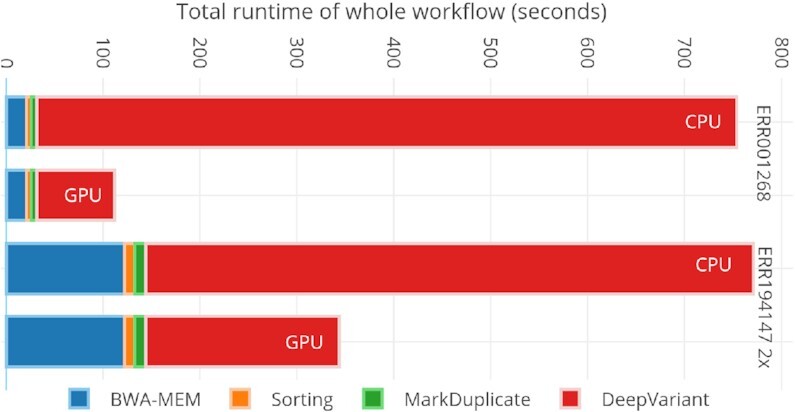
Total runtime for DeepVariant-based complete variant-calling workflow (VC@Scale), which uses best performance combination of nodes. For both datasets pre-processing (BWA-MEM, sorting, and MarkDuplicate) uses 16 nodes while 32 nodes are used for DeepVariant.

**Figure 10:**
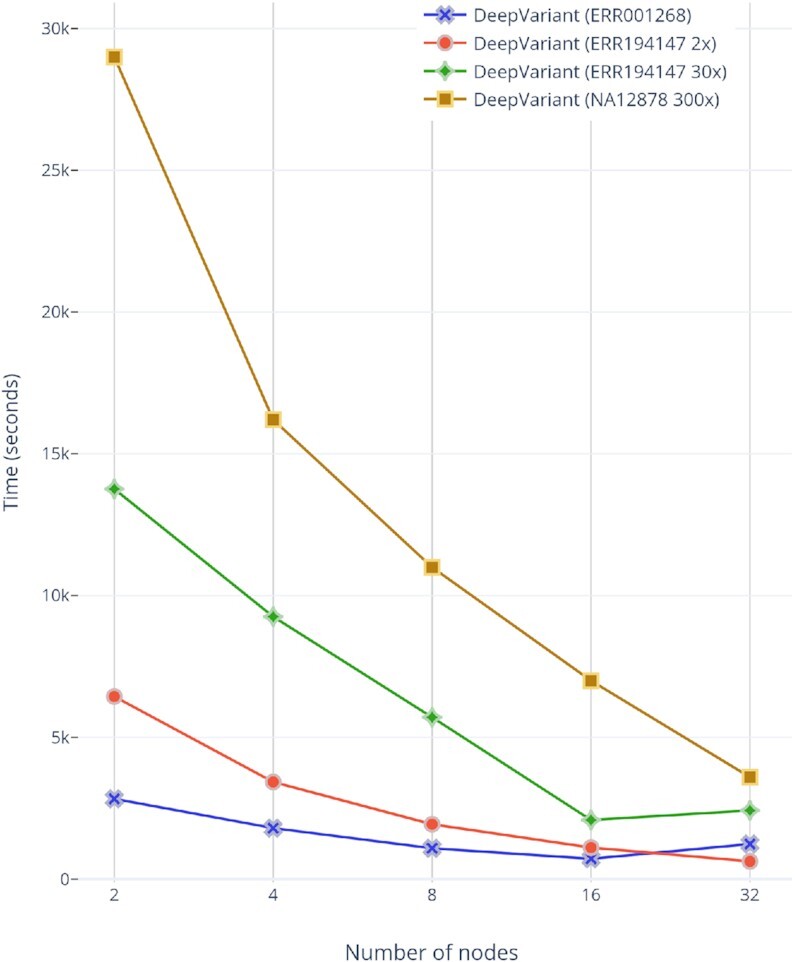
VC@Scale-DeepVariant scalability for different datasets and the number of nodes used in each run.

**Figure 11:**
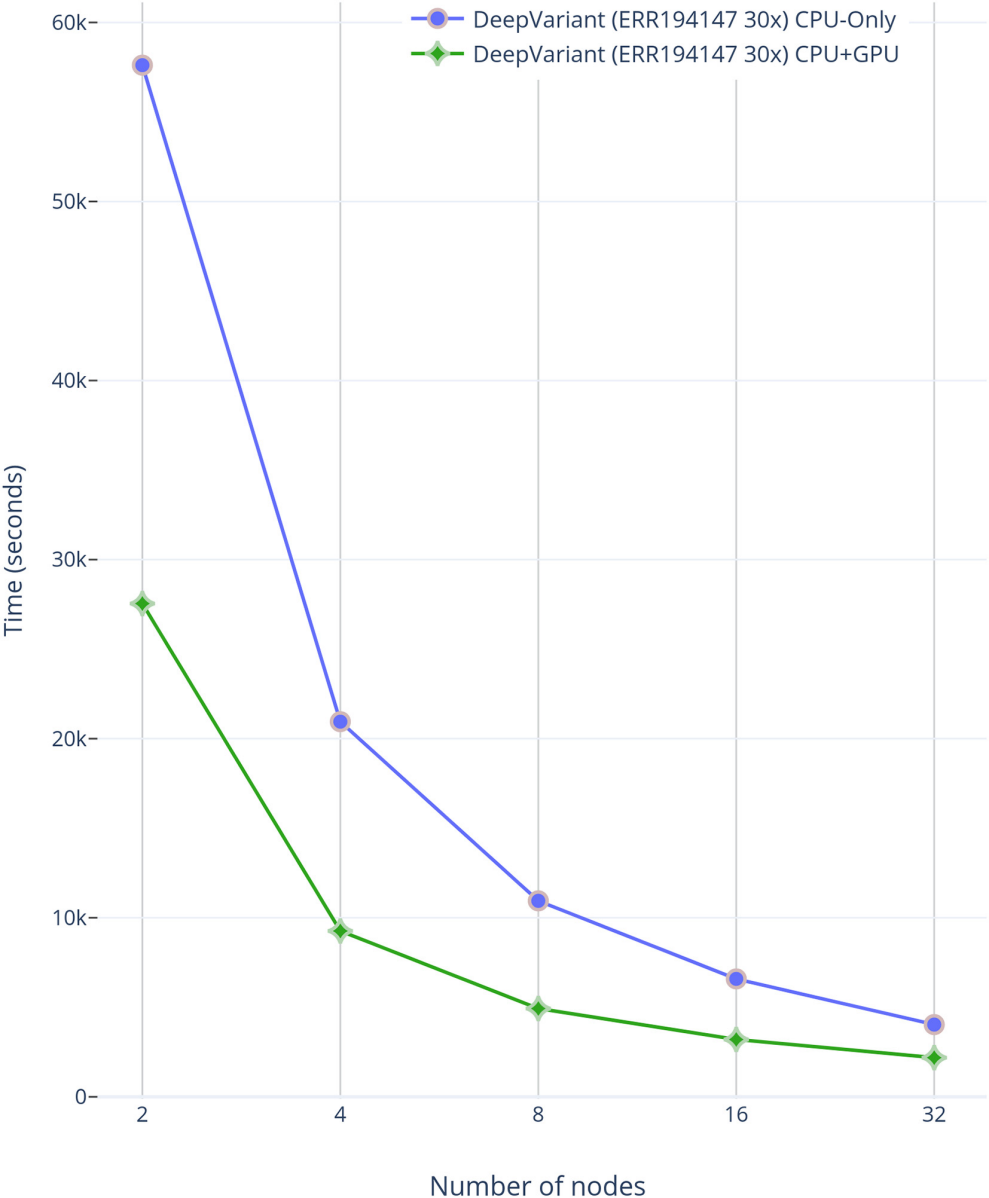
GPUs accelerated VC@Scale-DeepVariant scalability for ERR194147 (30×) dataset.

DeepVariant is ∼3–4 times slower than GATK’s HaplotypeCaller on CPU-only machines [47]. To make it scalable for clusters, we run each chromosome region independently on a different Spark worker node. In our pre-processing stage, we already store the load-balanced BAMs as individual chromosome regions. This approach provides a fruitful base for a subsequent variant-calling stage (DeepVariant in our case). For DeepVariant CPU-only version, we used a CPU cluster with different numbers of nodes (2, 4, 8, 16, and 32) and with multiple datasets such as ERR001268, ERR194147 (2×), ERR194147 (30×), and NA12878 (300×). Fig. 8 shows both CPU and GPU accelerated DeepVariant runtime while Fig. 9 shows the total runtime of variant calling workflow based on this work. In Fig. 10, the results show an increasing speed-up for DeepVariant scalability on a Spark cluster. In DeepVariant some smaller datasets perform best with just 16 nodes, while the processing trend of other datasets shows even more scalability when we increase the number of nodes from 16 to 32. The total runtime is decreased up to 8× as compared to a single CPU machine. DeepVariant consists of 3 steps: (i) make_examples, (ii) call_variants, and (iii) postprocess_variants. The first 2 steps are the most time consuming (see Fig. 1). To improve their performance, the make_examples step is multi-threaded for reading inputs and creating examples, while call_variants has been accelerated for GPUs. As shown in Fig. 8, we have observed in some datasets such as ERR194147 (30×) that the call_variants step takes up to 95% of the total time of DeepVariant. This step can be accelerated on GPUs by almost 10 times as shown in the GPU accelerated results of Fig. 8. Such acceleration makes it more feasible to adopt DeepVariant in practice. We also use a GPU cluster to test our approach for DeepVariant scalability, as well as acceleration. Results in Fig. 11 show >2 times speed-up with GPU accelerated DeepVariant for the ERR194147 (30×) dataset as compared to CPU-only.

### Variant-calling workflow

The total runtime results for the whole variant-calling workflow using BWA-MEM, Sorting, MarkDuplicate, and DeepVariant are shown in Fig. 9. Here we show the best possible node configuration for both the pre-processing and variant-calling stages. For the dataset ERR194147 (2×), in pre-processing the best fit is found with 16 nodes while 32 nodes give better scalability in variant calling. Similarly for dataset ERR001268, the choice of 16 nodes provides the best performance and scalability. The total runtime is decreased by up to 5 times as compared to a single CPU machine.

### Stand-alone BWA-MEM and DeepVariant

Our workflow can also be used as 2 independent components: a stand-alone BWA-MEM and a stand-alone DeepVariant component. The BWA-MEM component represents the fastest stand-alone Spark-based scalable implementation compared to other state-of-the-art BWA-MEM cluster solutions. In this solution we achieve almost linear speed-ups with increasing the number of nodes. The output is saved into separate SAM files, which can be merged through Samtools to output a single SAM file.

In this solution, an already created BAM file can be used with DeepVariant for variant calling on a cluster. As discussed in the Section “DeepVariant,” we used Samtools to split the BAM file into our pre-defined chromosome regions to generate load-balanced chromosome region parts. In this way we ran DeepVariant instances on Spark worker nodes. The output speed-up and scalability results are the same as described in Section “Variant calling (DeepVariant).”

### Standalone pre-processing (Piped)

#### BWA-MEM, Sambamba (sorting, markdup) and Samtools (merge)

This is a simple and efficient implementation of pre-processing stages (alignment, sorting, and markduplicate) on a Spark cluster. We integrated already existing and widely used tools in this workflow. Sambamba sorting and MarkDuplicate algorithms produce the same output as Picard’s. In this approach, the master node streams the FASTQ data to all worker nodes as discussed in Section “FASTQ chunks streaming.” All worker nodes initiate 1 BWA-MEM instance. The BWA-MEM output is then piped into Sambamba, which performs both SAM to BAM conversion and sorting. The Sambamba MarkDuplicate stage is optional. After these stages, we use the Samtools merge algorithm to combine all the resultant BAM files into a single BAM file. We have developed a demo with different nodes on a Google GCP DataProc cluster, which is publicly available and can be tested with GCP. A complete guide to executing this workflow is available on our project GitHub page [48].

### Support/integration of other variant callers

Any variant caller that can support region-specific variant calling can be integrated into this workflow. We integrate Octopus [23], a recently developed, accurate, and fast variant caller, as a use case to demonstrate the feasibility of integrating other variant callers in this approach. We also performed a comparison on DeepVariant and Octopus on Chr20-HG003 Illumina WGS reads publicly available from the PrecisionFDA Truth v2 Challenge, and we found that the accuracy of Octopus was almost identical to that of DeepVariant for both SNP and indel variants. We also provide a guide to reproduce both these use cases on GitHub.

## Discussion

Here we discuss some of the advantages and limitations of our approach, in addition to the advantages of using Apache Arrow as a common in-memory data format for variant-calling workflows.

### Portability of the implementation

The workflow implementations discussed in this article are portable to many HPC cluster environments. We use standard cluster solutions such as the Singularity container and the Slurm Workload Manager to deploy and reproduce them with ease on other cluster environments.

### Accuracy

To compare the detection accuracy of small variants in both single-node (default) method and VC@Scale (distributed) method, we used the HG002 (NA24385 sample with 50× coverage taken from PrecisionFDA challenge V2) dataset to detect SNP and indel variants using DeepVariant (v1.1.0), against the GIAB v4.2 benchmark set for HG002 dataset. The GA4GH small-variant benchmarking tool hap.py [49] has been used to compare the resulting variants in both methods. Tables 2 and 3 list the accuracy analysis results in terms of recall, precision, and F1-score. The tables show that in general VC@Scale has very comparable accuracy results to the baseline. Detailed inspection of the results shows that VC@Scale detects the same number of indel true-positive and false-negative results and slightly fewer false-positive results compared to the baseline. This gives the same recall results but ensures a slightly improved precision and F1-score. For SNPs, however, VC@Scale detects slightly fewer true-positive results but more false-negative and false-positive results. This gives a marginally degraded SNP recall, precision, and F1-score.

**Table 2:** Accuracy evaluation of small variants of HG002 (NA24385 with 50× coverage taken from PrecisionFDA challenge V2 datasets) against GIAB HG002 v4.2 benchmarking set for Chr1 on a single-node (default) run

Variant type	Truth total	True positive	False negative	False positive	Recall	Precision	F1-Score
Indel	42,689	42,390	299	131	0.992996	0.997053	0.995020
SNP	264,143	262,367	1,776	351	0.993276	0.998665	0.995963

**Table 3:** Accuracy evaluation of small variants of HG002 (NA24385 with 50× coverage taken from PrecisionFDA challenge V2 datasets) against GIAB HG002 v4.2 benchmarking set for Chr1 on a cluster-scaled (distributed) VC@Scale implementation

Variant type	Truth total	True positive	False negative	False positive	Recall	Precision	F1-Score
INDEL	42,689	42,390	299	127	0.992996	0.997142	0.995065
SNP	264,143	262,365	1,778	355	0.993269	0.998649	0.995952

Chr1 has been chunked into 10 parts. HG002-NA24385 datasource is available at https://precision.fda.gov/challenges/10.

### Parallelization and scalability

Owing to dividing chromosomes on the basis of regions for load-balancing in the alignment stage, better parallelization is achieved per node in both pre-processing and variant-calling stages. In the examples in this article, we created a total of 65 such regions, which allows us to scale up to 32 nodes for the pre-processing and DeepVariant stages. When using 32 nodes, 2 regions are being mapped to each worker node. The total runtime of the workflow is determined by the slowest node in the cluster. As the size of the input dataset increases, making smaller regions can give more scalability for higher number of nodes.

Two points are important to understand the scalability and performance predictability of such applications when using the Apache Spark framework. (i) Spark always takes some fraction of time to initialize the underlying processes on its worker nodes and also spends a similar amount of time in scheduling and collecting the result. Therefore, increasing the number of nodes Spark uses also increases this overhead time. If increasing the number of nodes results in a small overall processing time, then it reaches a point where the aforementioned overhead time surpasses the processing time. (ii) Data size also influences the scalability and performance of these applications, and this is directly linked to our previous point. When we increase the number of nodes, the data size is always divided by the number of nodes being used. So we have to figure out the best possible scenario of performance on the cluster when choosing the number of nodes and data size being used.

### System resource utilization

**Figure 12:**
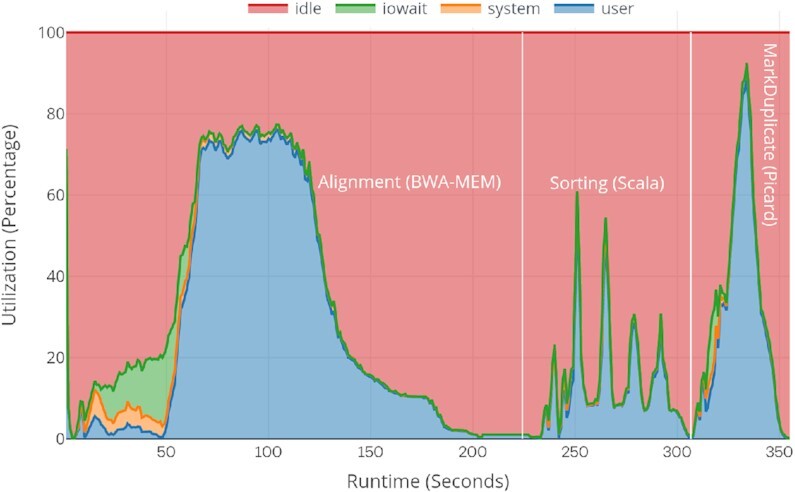
SparkGA2 cluster-wide system resource utilization graph for pre-processing stages.

**Figure 13:**
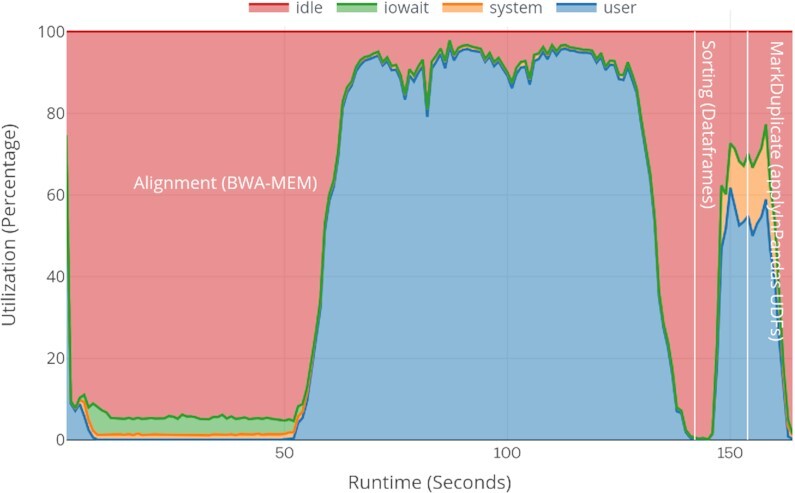
VC@Scale cluster-wide system resource utilization graph for pre-processing stages.

Existing Spark-based variant-calling workflows like ADAM, SparkGA2, and Halvade launch multiple instances of BWA-MEM on each Spark worker node, which degrades the actual performance of BWA-MEM instances on each individual node. These workflows store the output of each stage to the disk, which sometimes incurs I/O wait overheads, as well as reading and writing to I/Os for each stage, and parsing text SAM or compressed BAM also involves some additional overheads as shown in Fig. 12. The figure uses the ERR194147 (2×) dataset with a 16-node cluster (the best scalable and optimized use case for both SparkGA2 and in our approach). For comparison, we also show the system resource utilization for our approach in Fig. 13. In both approaches, the first 50 seconds are spent loading the FASTA index and reading the first FASTQ data chunk. In SparkGA2, the I/O wait time is a bit higher than ours because it loads multiple indices for multiple BWA-MEM instances on each node while we just load 1 FASTA index on each node. After loading the files, the actual alignment process starts. The figures show that in SparkGA2, a maximum of 78% of the CPU resources are being used for BWA-MEM while in our approach almost 95% on average of the CPU resources are being used for BWA-MEM. Similarly, in Sorting only ∼10% and in MarkDuplicate 50% on average CPU resources are being used in SparkGA2. In our approach, the timing graph shows that both stages can be almost completed in half of the total time with an average of 60–65% utilization. Because Spark uses lazy evaluations of Dataframes operations, we cannot distinguish exactly the timing for each operation separately. Owing to some internal shuffling and the PySpark to Pandas Dataframes conversion via Apache Arrow, slightly more system time is being spent there.

### Memory consumption

We use Plasma Object Store to place temporary BWA-MEM output data in-memory on each node. These objects are removed when the Spark Dataframes creation is accomplished. During this intermediate step we use a memory space that is twice the size of the SAM file. Similarly, during the sorting process, Spark does a lot of internal shuffling, which requires additional memory. In MarkDuplicate, we use Pandas UDFs, which internally use the Arrow data format for PySpark Dataframes to Pandas Dataframes conversion and vice versa. This step is also memory intensive. This workflow in pre-processing stages requires double the memory size as compared to SAM data produced by the BWA-MEM stage on that worker node while the master node requires a memory size equal to the total size of the SAM data produced by all worker nodes. For the DeepVariant stage, it only requires a couple of gigabytes of memory on both worker and master nodes.

## Conclusion

A scalable and high-performance DeepVariant-based variant-calling workflow for cluster-scaled environments is presented in this article. We use a FASTQ data streaming technique to feed data to an alignment stage followed by an in-memory data load-balancing method to store alignment output. Sorting and mark duplicate stages are implemented in such a way as to get benefits from the Apache Arrow data format. The load-balanced BAM file output of the pre-processing stages is used in DeepVariant, making variant calling more efficient on a compute cluster.

Scalability analysis of our approach shows significant reduction in runtime compared to a single node. For pre-processing stages, ERR001268 and ERR194147 (2×) datasets provide up to 7 and 8 times speed-up for 16 nodes, respectively. For DeepVariant, ERR001268 (1× coverage) gives 5 times, ERR194147 (2×) gives nearly 8 times, and ERR194147 (30×) and NA12878 (300×) give 12 times speed-up for 32 nodes as compared to single-node runtime. Similarly, our approach is faster than state-of-the-art workflows, such as SparkGA2, resulting in 1.8 and 2 times speed-up for ERR001268 (1×) and ERR194147 (2×) for pre-processing stages on 16 nodes, respectively. Our architectural approach also increases efficient system resource utilization. For pre-processing stages, we achieve 20%–25% better processor utilization, which in turn helps to speed up overall processing. The variant accuracy analysis on PrecisionFDA V2 challenge datasets against the GIAB truth v4.2 benchmark truth data shows almost identical results as compared to single-node runs. We also show the flexibility of this approach to adopt other variant callers. We integrate the Octopus variant caller as a use case for this purpose. We also demonstrate the deployment of this approach on public clouds; currently, Google GCP DataProc cluster has been used for this purpose.

## Availability of Source Code and Requirements

- Project name: VC@Scale (Scalable Variant Calling)
- Project home page: https://github.com/abs-tudelft/variant-calling-at-scale
- Operating system(s):  Platform independent
- Programming language: Bash, Python, C, C++
- Other requirements: Singularity, Apache Spark 3.0.1, Apache Arrow 3.0.0
- License: Apache 2.0
- biotools:variant-calling-at-scale

## Data Availability

Human Reference Genome, Build 37, is available for download (GRCh37/hg19) [43]. Illumina HiSeq-generated WGS paired-end read data of NA12878 with sample ERR001268 [40], Illumina HiSeq 2000 paired-end NA12878 with sample ERR194147 [41] with sequencing coverage of 30×, and Illumina HiSeq 300× HG002 sample of NA12878 [42] were used to evaluate this work and are publicly available. An archival snapshot of the code and supporting data is available via the *Gigascience* database, GigaDB [50].

## Abbreviations

API: Application Programming Interface; BAM: Binary Alignment/Map; BWA: Burrows-Wheeler Aligner; CNV: copy number variation; CPU: central processing unit; DF: Dataframe; GATK: Genome Analysis Toolkit; Gb: gigabase pairs; GPU: graphics processing unit; HDFS: Hadoop Distributed File System; HPC: high-performance computing; indels: insertions and deletions; I/O: input/output; JVM: Java Virtual Machine; kb: kilobase pairs; MPI: Message Passing Interface; NFS: Network File System; NGS: next-generation sequencing; RB: RecordBatches; RDD: resilient distributed datasets; SAM: Sequence Alignment/Map; SNP: single-nucleotide polymorphism; SV: structural variant; UDF: user-defined function; UPC: Unified Parallel C; VC@Scale: Scalable Variant Calling; VCF: Variant Calling File; WGS: whole-genome sequencing.

## Ethical Approval

All work is carried out with publicly available and authorized human genome datasets.

## Competing Interests

The authors declare that they have no competing interests.

## Funding

The PhD research of Tanveer Ahmad is generously funded by Punjab Educational Endowment Fund (PEEF), Pakistan.

## Authors' Contributions

Z.A.A and H.P.H. conceived and supervised this work. T.A. designed and developed the whole variant-calling workflow. All authors read and approved the final manuscript.

## Supplementary Material

giab057_GIGA-D-21-00032_Original_Submission

giab057_GIGA-D-21-00032_Revision_1

giab057_GIGA-D-21-00032_Revision_2

giab057_GIGA-D-21-00032_Revision_3

giab057_Response_to_Reviewer_Comments_Original_Submission

giab057_Response_to_Reviewer_Comments_Revision_1

giab057_Response_to_Reviewer_Comments_Revision_2

giab057_Reviewer_1_Report_Original_Submission Medhat Mahmoud -- 2/21/2021 Reviewed

giab057_Reviewer_1_Report_Revision_1 Medhat Mahmoud -- 6/19/2021 Reviewed

giab057_Reviewer_2_Report_Original_Submission Jan Fostier -- 2/23/2021 Reviewed

giab057_Reviewer_2_Report_Revision_1 Jan Fostier -- 6/23/2021 Reviewed

giab057_Reviewer_3_Report_Original_Submission Ted Ahn -- 3/5/2021 Reviewed

giab057_Reviewer_3_Report_Revision_1 Ted Ahn -- 6/18/2021 Reviewed

giab057_Supplemental_File
