# Supplementary material for: VC@Scale: Scalable and high-performance variant calling on cluster environments
Source: Gigascience. 2021 Sep 7;10(9):giab057. doi: 10.1093/gigascience/giab057 (PMC8424057; doi:10.1093/gigascience/giab057)
Supplement: giab057_GIGA-D-21-00032_Original_Submission [file giab057_giga-d-21-00032_original_submission.pdf]

GigaScience

SVCall: Scalable DeepVariant based Variant Calling on Spark cluster using Apache Arrow

--Manuscript Draft--

**Manuscript Number:**	GIGA-D-21-00032
**Full Title:**	SVCall: Scalable DeepVariant based Variant Calling on Spark cluster using Apache Arrow
**Article Type:**	Technical Note
**Funding Information:**	PEEF (2017) — Mr. Tanveer Ahmad
**Abstract:**	Background: In the past couple of years many new deep learning based variant calling methods like DeepVariant and NeuSomatic have emerged as more accurate methods as compared to conventional variant calling algorithms like GATK HaplotypeCaller/Mutect2, albeit at higher computational costs. Therefore, there is a need for more scalable and higher performance workflows of these deep learning methods. Almost all existing cluster scaled variant calling workflows that use Apache Spark/Hadoop as big data frameworks loosely integrate existing single node pre-processing and variant calling applications. Using Apache Spark just for distributing/scheduling data among loosely coupled applications or using I/O based storage for storing intermediate applications output does not exploit the full benefit of Apache Spark in-memory processing. In order to achieve this, we propose a native Spark-based workflow that uses Python and Apache Arrow to enable efficient transfer of data between different workflow stages. This benefits from the ease of programmability of Python and the high efficiency of Arrow's columnar in-memory data transformations.<br>Results: Here we present a scalable, parallel and efficient implementation of next generation sequencing data pre-processing and variant calling workflows. Our design tightly integrates most pre-processing workflow stages, using Spark built-in functions to sort reads by coordinates, and mark duplicates efficiently. Our approach outperforms state-of-the-art implementations by more than 2x for the pre-processing stages, creating a scalable and high performance solution for DeepVariant for both CPU-only and CPU+GPU clusters.<br>Conclusions: We show the feasibility and easy scalability of our approach to achieve high performance and efficient resource utilization for variant calling analysis on HPC clusters using the standardized Apache Arrow data representations. All codes, scripts and configurations used to run our implementations are publicly available and open sourced.
**Corresponding Author:**	Tanveer Ahmad<br>TU Delft: Technische Universiteit Delft<br>Delft, NETHERLANDS
**Corresponding Author Secondary Information:**	
**Corresponding Author's Institution:**	TU Delft: Technische Universiteit Delft
**Corresponding Author's Secondary Institution:**	
**First Author:**	Tanveer Ahmad
**First Author Secondary Information:**	
**Order of Authors:**	Tanveer Ahmad<br>Zaid Al Ars<br>Peter Hofstee
**Order of Authors Secondary Information:**	
**Additional Information:**	

Powered by Editorial Manager® and ProduXion Manager® from Aries Systems Corporation

Question	Response
Are you submitting this manuscript to a special series or article collection?	No
**Experimental design and statistics**<br><br>Full details of the experimental design and statistical methods used should be given in the Methods section, as detailed in our Minimum Standards Reporting Checklist. Information essential to interpreting the data presented should be made available in the figure legends.<br><br>Have you included all the information requested in your manuscript?	Yes
**Resources**<br><br>A description of all resources used, including antibodies, cell lines, animals and software tools, with enough information to allow them to be uniquely identified, should be included in the Methods section. Authors are strongly encouraged to cite Research Resource Identifiers (RRIDs) for antibodies, model organisms and tools, where possible.<br><br>Have you included the information requested as detailed in our Minimum Standards Reporting Checklist?	Yes
**Availability of data and materials**<br><br>All datasets and code on which the conclusions of the paper rely must be either included in your submission or deposited in publicly available repositories (where available and ethically appropriate), referencing such data using a unique identifier in the references and in the "Availability of Data and Materials" section of your manuscript.	Yes

Have you have met the above requirement as detailed in our Minimum Standards Reporting Checklist?	

Powered by Editorial Manager® and ProduXion Manager® from Aries Systems Corporation

PDF of manuscript Click here to access/download;Manuscript;SVCall.pdf

GigaScience, 2020, 1–12

doi: xx.xxxx/xxxx
Manuscript in Preparation
Technical Note

TECHNICAL NOTE

SVCall: Scalable DeepVariant based Variant Calling on Spark cluster using Apache Arrow

Tanveer Ahmad[1,*], Zaid Al Ars[1] and H. Peter Hofstee[1,2]

[1]Delft University of Technology, Delft, Netherlands and [2]IBM Austin, Texas, USA

[*]t.ahmad@tudelft.nl

Abstract

Background: In the past couple of years many new deep learning based variant calling methods like DeepVariant and NeuSomatic have emerged as more accurate methods as compared to conventional variant calling algorithms like GATK HaplotypeCaller/Mutect2, albeit at higher computational costs. Therefore, there is a need for more scalable and higher performance workflows of these deep learning methods. Almost all existing cluster scaled variant calling workflows that use Apache Spark/Hadoop as big data frameworks loosely integrate existing single node pre-processing and variant calling applications. Using Apache Spark just for distributing/scheduling data among loosely coupled applications or using I/O based storage for storing intermediate applications output does not exploit the full benefit of Apache Spark in-memory processing. In order to achieve this, we propose a native Spark-based workflow that uses Python and Apache Arrow to enable efficient transfer of data between different workflow stages. This benefits from the ease of programmability of Python and the high efficiency of Arrow's columnar in-memory data transformations.
Results: Here we present a scalable, parallel and efficient implementation of next generation sequencing data pre-processing and variant calling workflows. Our design tightly integrates most pre-processing workflow stages, using Spark built-in functions to sort reads by coordinates, and mark duplicates efficiently. Our approach outperforms state-of-the-art implementations by more than 2x for the pre-processing stages, creating a scalable and high performance solution for DeepVariant for both CPU-only and CPU+GPU clusters.
Conclusions: We show the feasibility and easy scalability of our approach to achieve high performance and efficient resource utilization for variant calling analysis on HPC clusters using the standardized Apache Arrow data representations. All codes, scripts and configurations used to run our implementations are publicly available and open sourced.

Key words: Whole Genome Sequencing; Apache Spark; Apache Arrow; BWA-MEM; Sorting; MarkDuplicate; DeepVariant

Introduction

Immense improvements in Next Generation Sequencing (NGS) technologies enable producing large amounts of high throughput and cost-effective raw genome datasets. On the one hand, this development paves the way to analyze more genomes with higher accuracy, but at the same time this creates the computational challenge of processing such a large amount of data in a timely fashion. The approximate raw data size of the human genome sequenced using NGS technologies is 300 GB when sequenced with 30x coverage, and can be more than 1 TB raw data with 300x sequencing coverage. The ongoing pace of development of these technologies promises even longer reads of up to 100 kbp and with more coverage depth.

To process and prepare raw data for downstream analysis, many open-source and proprietary bioinformatics tools and workflow are available to run on single-node machines. But due to the continuous growth in genomics datasets, processing this data on a single node becomes inefficient and time consuming because of Input/Output bottlenecks, limitations on the number of physical cores in a single CPU and memory capacity constraints. To scale up these tools for distributed

Compiled on: January 28, 2021.
Draft manuscript prepared by the author.

Key Points

- Apache Spark based high performance and scalable pre-processing (alignment, sorting and duplicates removal) implementation in PySpark by leveraging the Apache Arrow in-memory data format.
- Scalable DeepVariant solution, for complete variant calling analysis on Apache Spark, and using the output of pre-processing stages.
- The most efficient cluster scaled implementation gives linear speedup by increasing the dataset size and number of nodes in the cluster.
- For paired-end reads, Picard MarkDuplicate compatible efficient algorithm is implemented in Python using Pandas UDFs.
- Compatible output formats for both BAM and VCF files, enables parallel and scalable further downstream analysis.
- Implementation techniques are tested and evaluated on Dutch National SurfSara Cartesius HPC infrastructure.
- We use 2, 4, 8, 16, and 32 nodes HPC clusters and similarly used different sequencing coverage datasets like, ERR001268, ERR194003 (30x) and HG002-NA24385 (300x) to analyze the scalability and performance of our methods.
- For DeepVariant scalability and performance analysis both CPU only and CPU+GPU cluster is used. GPU accelerated DeepVariant performs very well on call_variants step, giving up to 50x speedup.
- All the code and scripts to test/compare existing frameworks for the complete variant calling workflow are available as open source.

computing environments, both high performance computing (HPC) programming models (using MPI) and big data frameworks (using Hadoop and Spark) have been explored in the past decade.

MPI (message passing interface) implementations leverage the benefits of distributed memory architectures in inter-node communication. The workflow can exploit the maximum bare-metal performance of such multi-node clusters using shared memory MPI implementations. Previously, too little emphasis has been put on developing MPI based cluster scaled bioinformatics tools and workflows. The reason can be the lack of fault-tolerance [1], redundant data replication, and complexity to develop parallel algorithms in this approach. However, new fault-tolerance models for MPI [2] can enable fault-tolerance mechanisms for such applications and workflows. Similarly, the availability of one-sided communication in new MPI-3 RMA (Remote Memory Access) standard promises better performance gains in the applications while requiring no (or very little) inter-node data sharing and communication. Many tools in a variant calling workflow exhibit such property of not sharing data between the nodes and may run independently (with the exception of sorting).

Apache Hadoop [3] is a MapReduce framework used to process chunks of big datasets in parallel on large cluster nodes in a fault-tolerant and reliable manner. MapReduce usually splits the input data into smaller chunks, runs these chunks completely independently in map tasks, sorts the output of these tasks which is fed to a reduce task as input to generate the final output. MapReduce exclusively uses key-value pair input data to process, sort and aggregate the output based on keys. Hadoop Distributed File System (HDFS) is commonly used to store the input and output data on local compute nodes or on network storage nodes. Some early variant calling workflows like Halvade [4] use this approach to exploit computing cluster resources by running multiple legacy application instances (loosely integrated in the Apache Hadoop Framework) in parallel on chunked input data.

Apache Spark [5] is a unified analytics engine to process big data in a distributed computing environment, with built-in modules for streaming data, distributed machine learning, SQL functions and graph processing. Spark also provides high-level APIs for Java, Scala, Python and R languages. In Spark, resilient distributed datasets (RDDs) are the core components that are distributed across the nodes of a cluster to be operated on in parallel. RDDs can be cached/persisted in-memory across nodes to store intermediate results for iterative processing. Spark commonly uses HDFS to read/write data, but also supports other storage systems like NFS, HBase and Amazon's S3. Many variant calling workflows and tools have been developed over the last decade since its first release, including SparkGA2 [6], ADAM [7], SparkBWA [8], BWASpark [9], PipeBWA [10], etc.

In this article, we propose and implement a new framework that combines the advantage of easy programmability of Apache Spark and the high efficiency of MPI. The resulting framework integrates Apache Spark NGS data pre-processing with the Apache Arrow in-memory data format. Our framework tightly integrates pre-processing (reads sorting and duplicate removal) applications in Python using distributed Dataframes (DF) based sorting and vectorization. This is the first ever such implementation for genomics data to exploit the benefits of Apache Arrow in-memory data format in Apache Spark. The key contributions of our approach are as follows:

- The first scalable approach for DNA data pre-processing that uses Apache Arrow for efficiently utilizing compute resources while preserving easy programmability
- Improved performance of up to 2x compared to state-of-the-art scalability approaches
- Integration with DeepVariant to create the first scalable open source DeepVariant workflow on Apache Spark

This article is organized as follows. In Section "Background and related work", we discuss single node and cluster scaled pre-processing and variant calling workflows, followed by Section "Methods" which presents the in-depth details of the new Apache Arrow based data format for NGS data. In Section "Design and Implementation", the internal design flow and implementation details of our new efficient workflow are discussed. Furthermore, Section "Results and Evaluation" describes the results of our implementation using different nodes configurations with different sequencing coverage/depth datasets to show the scalability and the performance comparisons with state-of-the-art methods. In Section "Discussion", more detailed insights on performance, scalability, resources utilization and memory consumption are given. Finally, Section "Conclusion" ends with some concluding remarks and possible future directions.

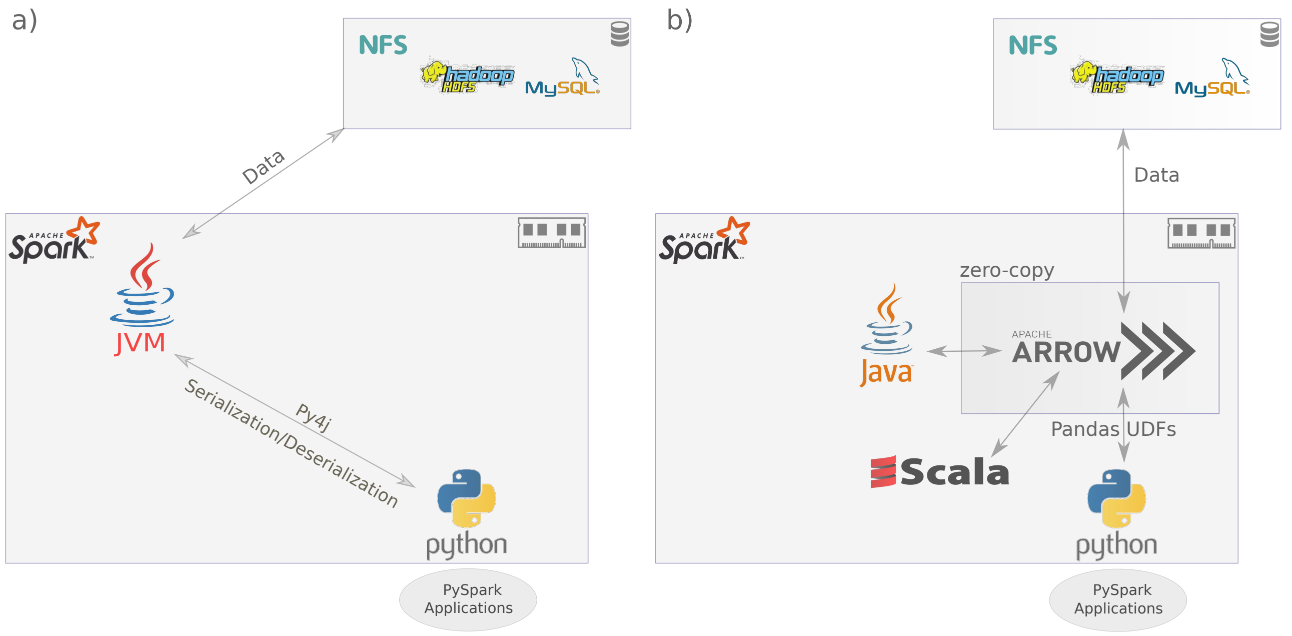

Figure 1. **a)** Python programs in Spark require inefficient data serialization/deserialization between Python and JVM processes (using the Py4j library). **b)** Efficient data communication using the new Apache Arrow unified in-memory columnar data format with zero-copy overhead for languages in Apache Spark.

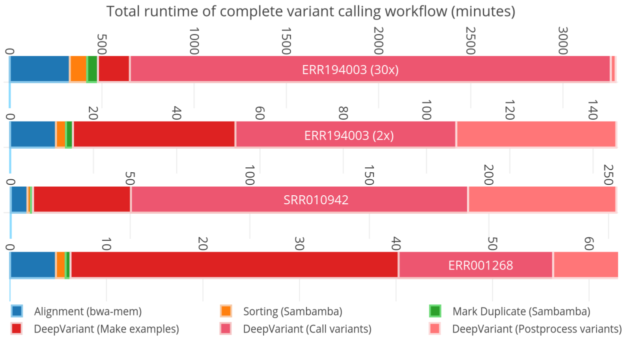

Figure 2. Single node total runtimes for complete variant calling workflow using DeepVariant for different datasets.

Background and related work

In this section, first we introduce and discuss some tools used to pre-process NGS data followed by a discussion of some widely used cluster scale variant calling workflows.

Pre-processing NGS data

Pre-processing of NGS data includes a number of steps: 1. alignment of raw FASTQ data against a reference genome, 2. chromosome based coordinate sorting, and 3. Polymerase Chain Reaction (PCR) duplicates removal. These steps are important in every variant calling workflow. There are many tools available publicly to pre-process NGS data efficiently on single node machines. Bowtie2 [11] and BWA-MEM [12] tools are widely used for short read sequence alignments, while tools like Minimap2, BLASR, GraphMap and DALIGNER [13] are being used for long reads alignment and mapping. SAMtools [14], Picard [15], Sambamba [16] and samblaster [17] are some of the most famous and widely used tools for the purpose of indexing, sorting, and duplicates removal in SAM/BAM/CRAM files.

Variant calling

Variant calling reveals deep insights into nucleotide-level organismal differences in some specific traits among populations from an individual genome sequence data. It discerns genetic variations in three categories like single nucleotide polymorphisms (SNPs), insertions and deletions (indels), and/or structural variants (SVs, may also include Copy Number Variations (CNVs), duplication, translocation, etc). The GATK HaplotypeCaller is a widely used variant caller to detect germline variations. Tools like VarScan [18], VarDict [19], MuTect2 [20] are used for somatic variant calling analysis. NeuSomatic [21, 22] is deep convolutional neural networks based somatic variant caller which runs in both standalone and ensemble modes (MuTect2, MuSE, Strelka2, SomaticSniper, VarDict, and VarScan2) for accurate somatic variants detection. FreeBayes [23], Strelka2 [24], DeepVariant [25], SNVer [26] and LoFreq [27] are also used for both germline and somatic variant calling analysis. The DeepVariant variant caller based workflow outperforms in both PrecisionFDA (pFDA) Challenges v1 [28] (highest SNP performance) and v2 [29] (all benchmark regions for PacBio and multi, difficult-to-map regions for ONT). DeepVariant does not required some additional pre-processing steps like base quality recalibration. Therefore we selected this variant caller to integrate with our pre-processing workflow. As shown in Figure 2, we run the fastest pre-processing tools with DeepVariant on a single machine with different datasets to get an idea of individual tool runtime in the workflow.

Cluster scaled workflows

There are many cluster scaled multi-node implementations available for alignment using both HPC languages like MPI/Unified Parallel C (UPC) as well as big data framework like Hadoop MapReduce and Apache Spark. pBWA [30] and mpiBLAST [31] use MPI and CUSHAW3 [32] uses UPC++. Similarly ADAM's Cannoli [7], SparkBWA [8] and PipeMEM [10] are a few Apache Spark based BWA implementations that use BWA as loosely integrated underneath these implementation while GATK BWASpark modifies the original BWA to exploit the Spark

Table 1. A comparison of NGS data pre-processing workflows with their output storage approaches for each stage.

Framework	Alignment (output)	Sorting (output)	Duplicates removal (output)
Halvade	*.SAM in disk	in-memory (elPrep)	in-memory (elPrep)
SparkGA2	*.fq.gz in disk	*.BAM in disk	*.BAM in disk
ADAM	ADAM Parquet in disk	ADAM Parquet in memory	ADAM Parquet in memory
SVCall (this work)	in memory (Apache Arrow RecordBatches)	in memory (PySpark DFs)	in memory (PySpark DFs -> *.BAM)

scheduling and shuffling functionality to run BWA instances in parallel on clusters.

ADAM, Halvade and SparkGA2 are few implementations that also handle whole variant calling workflows based on GATK best practices including alignment, sorting, duplicates removal and base quality score recalibration.

ADAM, Halvade and SparkGA2 use the built-in Scala API in Spark for sorting the aligned reads. As Picard MarkDuplicate algorithm is considered as standard for paired-end reads for duplicates removal, SparkGA2 and Halvade use this Picard MarkDuplicate tool in Spark for distributed processing on cluster while ADAM has implemented their own duplicates removal algorithm in Scala which is nearly identical to the Picard MarkDuplicate algorithm. A more detailed comparison of these workflows for each individual pre-processing stage output storage strategy is given in Table 1.

Apache Arrow in Apache Spark

Apache Arrow [33] is an in-memory standard columnar data format. Apache Arrow also provides API interfaces and functions to process datasets in Go, C, C++, C#, Java, JavaScript, R, Rust, MATLAB, Ruby and Python languages. Due to the columnar data storage, efficient vectorized data analytics operations and better cache locality can be exploited. This in-memory format also supports zero-copy reads for large datasets in inter-process communication without serialization/deserialization overheads. Figure 1 shows how a common Apache Arrow based data format is being used in Apache Spark with different language interfaces.

Apache Spark leveraging Apache Arrow [34]

In this paper, we use Python as the language to implement our workflow due to its high level of abstraction and ease of implementation. It also has a stable API to Apache Arrow used in Apache Spark to efficiently transfer data between JVM and Python processes.

Pandas user-defined functions (UDFs)

The Python computation model in PySpark on UDFs is scalar, i.e., during UDF evaluation, the JVM executor process sends row data to PySpark workers which invoke UDFs on a row-by-row basis and send the results back to the executor process. However, the current Spark/PySpark release uses immutable Arrow RecordBatches (RBs) data instead of Spark built-in row based data. This enables vectorized UDFs evaluation on these RBs using Pandas Dataframes, which in turn gives a huge performance improvement. Due to vectorized UDF operations, the reduced number of system calls enables faster I/Os.

As traditionally Apache Spark uses a row based memory layout, using Arrow RBs requires converting Spark row based data to Arrow RecordBatch and vice versa to apply vectorized UDF operations in Pandas Dataframes. Some other operations (like grouped data in Pandas Dataframes on UDFs, and converting Spark Dataframes to/from Pandas Dataframes) are also becoming more efficient using Arrow underneath, which is discussed in more details in Section "Methods".

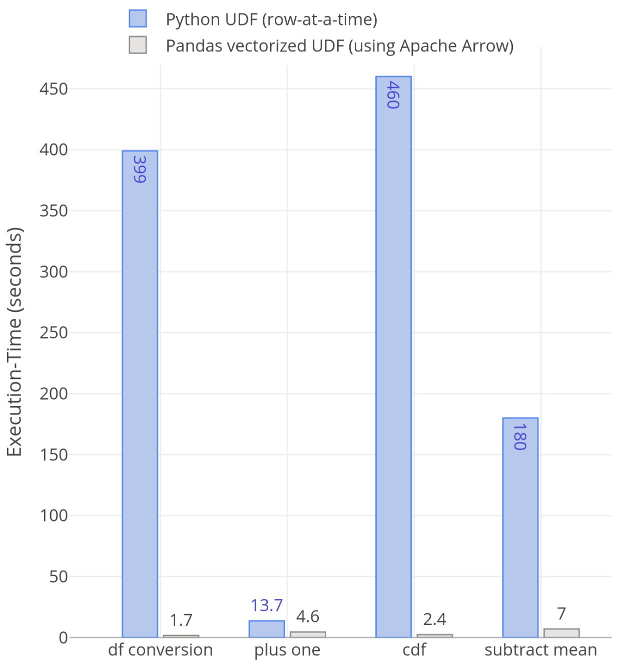

Figure 3. Performance comparison of Pandas dataframe to PySpark dataframe conversion using Arrow and without Arrow and Python UDF (row-at-a-time) and Pandas vectorized UDF (using Apache Arrow) operations: plus one, cdf and subtract mean.

Pandas function APIs

Python native functions can be applied on PySpark Dataframes, which input/output Pandas instances. Grouped map, map, cogrouped map are a few Pandas API functions to apply on PySpark Dataframes. These functions use Arrow to transfer data and Pandas to work on that data. These functions shares the same characteristics as those of Pandas UDFs.

UDF performance with/without Arrow

The Spark Python API supports UDFs which operate one-row-at-a-time, resulting in a large serialization and invocation overhead. Apache Arrow based unified memory format brings the benefits of high performance and low overhead dataframes conversion (PySpark<->Pandas) and vectorized Pandas UDFs operations in Python native environments. Because Spark inherently operates on row based memory layouts and Arrow data format is columnar which requires row-column conversions (Spark row <-> Arrow RecordBatch) overhead when doing these operations. In Figure 3, we show the performance comparison of 1) converting a Pandas dataframe to PySpark dataframe with Arrow and without Arrow, 2) Python UDF (row-at-a-time) and Pandas vectorized UDF (using Apache Arrow) for plus one, 3) cumulative probability distribution function (cdf), and 4) subtract mean examples [35].

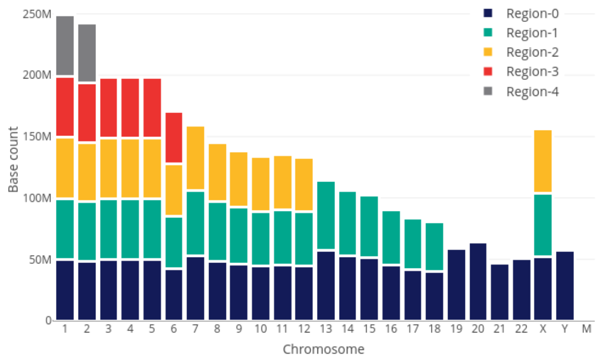

Figure 4. Static load balancing technique adopted in this work for BWA-MEM output which divides chromosomes based regions to join and process them in parallel for all further workflow stages.

Methods

In this section, we discuss the details of architectural approaches we have adopted in this work for processing the variant calling workflow.

Overview

The benefits of using distributed big data frameworks to process genomics data are fourfold: they provide easy and flexible deployment, efficient cluster scalability, fault-tolerance, as well as cheaper costs on public clouds and private HPC clusters. Traditionally, these frameworks use distributed file systems like the Hadoop distributed file system (HDFS) or the Network File System (NFS) for storage. The intermediate processing stages place data in-memory on-demand if enough memory is available in the form of RDDs. RDDs generally store data in their internal row format while Apache Arrow provides an efficient columnar data format to create distributed RDDs of Arrow RecordBatches object types.

To validate the scalability and performance advantage of our Apache Arrow based in-memory data placement, shuffling, conversion and computation techniques in Apache Spark using PySpark, we present the design methods for a full variant calling workflow. We have also developed high performance and scalable but very simple, portable and stand-alone methods for BWA-MEM and DeepVariant scalability on HPC clusters using traditional I/O based storage.

Variant calling workflow

In this subsection, we describe the various stages of the variant calling workflow that we designed, as shown in Figure 5. We start with the implementation of the pre-processing stages (alignment, sorting and duplicate removal) using Apache Arrow in-memory data format for temporary data storage in Plasma Stores, shuffling/conversion of data and transformations/computations on this data. The resultant data from these pre-processing stages is saved in BAM format. Each BAM file contains the reads of a particular chromosome and a specific region inside a chromosome. Variant caller (DeepVariant) instances process these BAM files on worker nodes and produce VCF files which are merged to produce a final VCF file.

FASTQ chunks streaming

We use the SeqKit [36] to create the FASTQ input chunks in parallel with BWA-MEM for input paired-end NGS data as shown in ❶ of Figure 5. SeqKit is an efficient multi-threaded utility, through which we provide this FASTQ data to BWA-MEM instances in streaming fashion, without the need to create FASTQ chunks separately. The number of created FASTQ chunks can be configured in the options parameters, depending on the number of nodes available in the Spark cluster.

Arrow integration in BWA-MEM

BWA-MEM is the most popular alignment tool in the bioinformatics community due to its efficient and accurate alignment algorithm for short reads. In our implementation, each Spark cluster worker node runs one BWA-MEM instance as shown in ❷ of Figure 5. We have modified BWA-MEM to output in-memory key-value pair SAM data instead of creating tab-delimited SAM files.

Key-value pairs

Key-value pair based data has proven efficient sorting performance as compared to text/columnar data structures. For every read, after creating its respective SAM fields we convert the whole read SAM data into a key-value pair `<POS:SAM>` and with `RNAME`, an extra information in the structure to store it in a designated immutable Arrow RecordBatch. Each RecordBatch is a combination of a schema, which specifies the types of data fields and the data itself. In our case, `POS` field is integer (Int) type while `SAM` and `RNAME` fields are String type.

Static load balancing

Due to the size differences in the chromosomes of the human genome, we created chromosomes regions for efficient scalability in BWA-MEM and the same such trend is followed in subsequent pre-processing stages as well. The number of regions is different for each chromosome to store reads corresponding to their respective regions as shown in Figure 4. Each region in each chromosome is on average equal to 40-50 million bps.

Plasma Object Store

The Plasma Object Store is an inter-process communication (IPC) component of Apache Arrow that handles shared memory pools across different heterogeneous systems [37]. To perform IPC, processes can create Plasma objects inside the shared memory pool that are typically data buffers underlying an Arrow RecordBatch. We cannot use more than half of overall system memory for these Plasma Stores. Through the shared memory pool, Plasma enables zero-copy data sharing between the processes. The output SAM data from BWA-MEM instances on each node is being stored in key-value pairs in respective chromosomal regions using the Arrow in-memory format as shown in ❸ of Figure 5.

flatMap() on BWA-MEM

We apply the PySpark `flatMap()` function on BWA-MEM instances which use an already SparkContext parallelized/distributed collection of input FASTQ chunks described in Section "FASTQ chunks streaming". All the BWA-MEM instances create Arrow RecordBatches of regions individual chromosomes on their own respective nodes. These Batches are temporarily placed in Plasma Object Stores on each node.

RDDs of Arrow RecordBatches

As soon as the alignment process on Apache Spark worker nodes finishes, we create distributed RDDs of these Arrow RecordBatch objects available across all the Spark worker nodes as shown in ❹ of Figure 5. Each RDD occupies the RecordBatches of a particular chromosome (with its specific region) distributed among all the worker nodes. Arrow RecordBatches

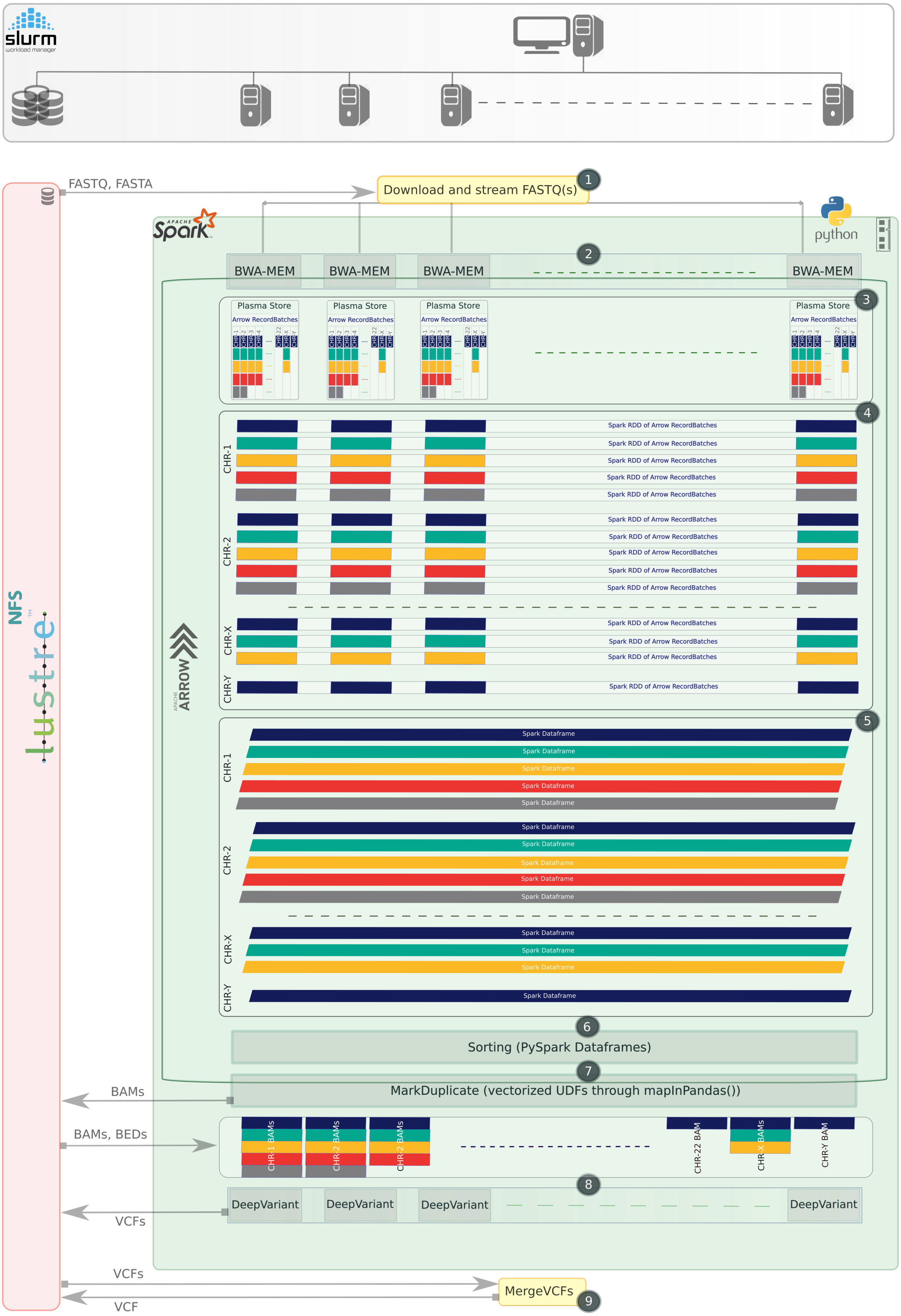

Figure 5. Complete design flow of the variant calling workflow implementation in SVCall, this design encompasses Lustre as file system, Apache Arrow as in-memory data format for pre-processing and DeepVariant as variant caller.

are filtered out in this step and cached into the Spark context of the master node.

RDDs to Dataframe

These RecordBatches in RDDs are serialized and a PySpark schema is generated through corresponding Arrow schema enclosed in these RecordBatches. Python objects to Java object conversion on RDDs is then applied as shown in ❺ of Figure 5. Finally, these resultant RDDs are converted to Spark Dataframe through Scala `PythonSQLUtils` methods. At this point, we have distributed Spark Dataframes of specific regions of each chromosome. We process these specific chromosomes regions independently and in parallel in the next sorting and duplicate removal stages.

Sorting

All the Spark Dataframes containing specific chromosome regions are sorted (❻ Figure 5) by coordinates through `df[n].orderBy('beginPos', ascending=True)` function. This function is very fast and efficient in sorting huge distributed Dataframes. All the Dataframes are sorted in parallel using the Python multiprocessing library `Pool` method.

Duplicates removal

Duplicate removal algorithms in this implementation were written from scratch in Python for both single and paired-end reads. These algorithms are developed using Pandas UDFs to apply on PySpark Dataframes which can use the Pandas function APIs (`df[n].groupby().applyInPandas()`) to leverage the benefits of Arrow for data transfer/conversion and transformations (❼ Figure 5). For paired-end reads, a Picard MarkDuplicate compatible algorithm has been developed. The accuracy of this algorithm is validated using different datasets, so that they can be used as a cluster scalable replacement for the existing Picard MarkDuplicate algorithm.

DeepVariant integration

DeepVariant is a being considered as an accurate variant caller for detection of both SNPs and indels in germline and somatic datasets. Their published results show that DeeVariant performs best for most PrecisionFDA Truth Challenge datasets [38]. We have observed that on a single node, DeepVariant scales very well up to 6-12 threads.Therefore we have enabled running multiple DeepVariant instances on each Spark worker node using the PySpark `flatMap()` function (❽ Figure 5). Each of DeepVariant instance takes input BAM (and BED as well in case of WES data) and reference FASTA from the I/O based NFS and produces individual VCF/gVCF files.

VCFs merge

Finally, the individual VCFs/gVCFs created through DeepVariant instances are merged (❾ Figure 5) through `Samtools` to produce a final complete VCF/gVCF file(s) for further downstream analysis.

Standalone implementations

In addition to implementing the complete workflow, we can also use BWA-MEM and DeepVariant as scalable stand-alone implementations capable of scaling almost linearly on HPC clusters depending on the input data size and number of nodes available.

BWA-MEM

Almost all BWA-MEM cluster scaled implementations (SparkBWA [8], BWASpark [9], PipeMEM [10], ADAM [7], and SparkGA2 [6]) run multiple BWA-MEM instances on each Spark worker node as Spark tasks, which degrades the underlying efficient single node multi-threaded scalability of this tool. Instead we use one BWA-MEM instance on each Spark worker node, storing output SAM files on storage and merging these SAM files to generate a single output SAM file.

DeepVariant

We use Samtools to generate different BAM files representing chromosome regions from a single BAM file in accordance with our human chromosome regions based approach as discussed earlier in Section "Static load balancing". Similarly, we have divided the reference FASTA into individual chromosome based FASTA files using faSplit [39]. The VCF/gVCF output files of these instances can be merged through Mergevcf or Samtools.

Results and evaluation

In this section, first we shortly describe the datasets and HPC infrastructure used in the evaluation of our techniques. In addition, we compare our results with other state-of-the-art frameworks for both pre-processing and variant calling stages followed by a detailed analysis and comparison of scalability, performance and speedups with these frameworks.

Datasets

We use multiple whole genome sequencing datasets with varying coverage depth to analyze the maximum possible scalability and performance of our methods. The first dataset is sample ERR001268 from the 1000 Genomes Project (phase 3) Illumina HiSeq generated WGS paired-end read data of NA12878 [40]. In addition, we used Illumina HiSeq 2000 paired-end NA12878 cell line data sequencing sample ERR194003 [41] with sequencing coverage of 30x. We also used 300x sequencing coverage WGS data from Genome in a Bottle (GIAB) aligned with novoalign for the Illumina HiSeq 300x reads for NA12878 [42] to analyze the scalability of DeepVariant. Human Genome Reference, Build 37 (GRCh37/hg19) [43] is used as a reference genome.

Evaluation HPC cluster

All experiments and comparisons are performed on the SurfSara Cartesius [44] HPC cluster (part of the Dutch national supercomputing infrastructure). Each CPU-only node is equipped with a dual socket Intel Xeon Processor (E5-2695 v2 or E5-2690 v3) running at 2.4/2.6GHz. Each processor has 12 physical cores with support of 24 hyper-threading jobs. Similarly, each CPU+GPU node is equipped with a dual socket Intel Xeon Processor (E5-2450 v2) running at 2.5GHz and 2x NVIDIA Tesla K40m GPGPUs. Each processor has 8 physical cores with support of 16 hyper-threading jobs. A total of 64-GBytes (E5-2695 v2/E5-2690 v3) and 96-GBytes (E5-2450 v2) of DDR4 DRAM with a maximum of 59.7 GB/s bandwidth is available for the whole system. A local storage of 1-TBytes and the same amount of network attached storage is available on the system. All nodes are connected through Mellanox ConnectX-3 or Connect-IB InfiniBand adapter.

Lustre [45] distributed and parallel file system is attached to our evaluation HPC cluster. Lustre file system has similar per-

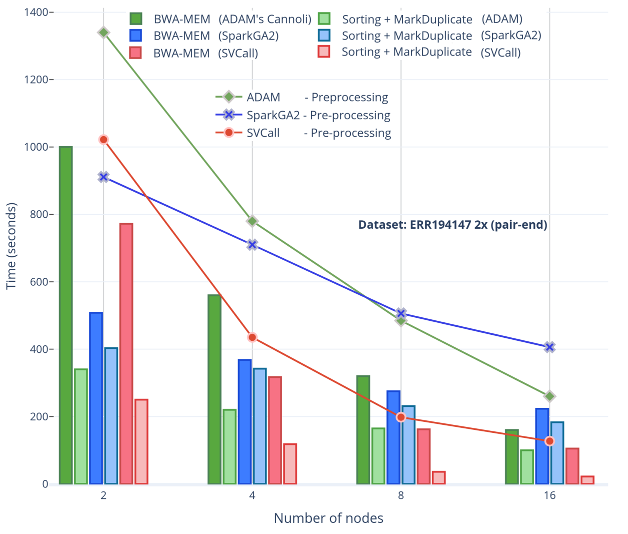

Figure 6. SVCall, SparkGA2 and ADAM comparisons of scalability for pre-processing stages using different number of nodes for ERR194003 (2x) dataset.

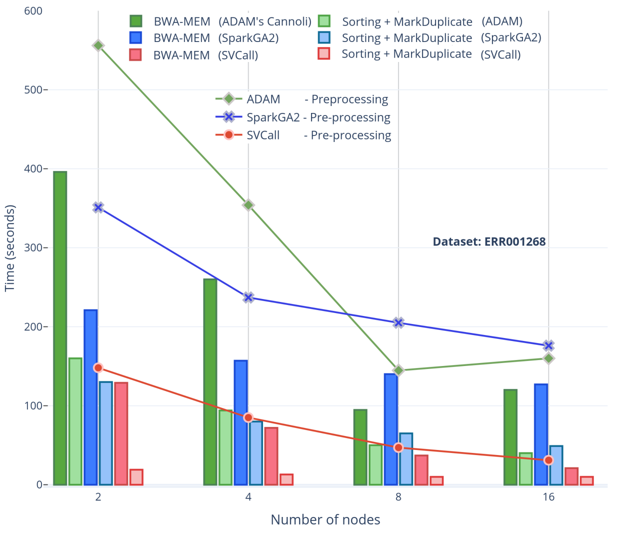

Figure 7. SVCall, SparkGA2 and ADAM comparisons of scalability for pre-processing stages using different number of nodes for ERR001268 dataset.

formance as of HDFS/YARN-based Hadoop cluster for shuffle-heavy workloads in Apache Spark.

Red Hat Enterprise Linux operating system is installed on all nodes. Apache Spark cluster is created in deploy-mode 'client' thorough Slurm [46] Workload Manager and all workflows are executed through bash scripts.

Pre-processing (BWA-MEM, Sorting, Duplicates removal)

Our approach performs pre-processing in a more tightly coupled fashion (i.e., using native PySpark functions) as compared to alternative solutions such as SparkGA2 which stores the output of each of the pre-processing stages to storage and loads it again for subsequent stages. We have tested the scalability and performance of our architectural choices with that of SparkGA2 and ADAM for different cluster sizes; 2, 4, 8 and 16 nodes have been used in almost all comparisons. Storing BWA-MEM output to in-memory key-value pairs using the Arrow format involves almost zero cost overhead for loading data

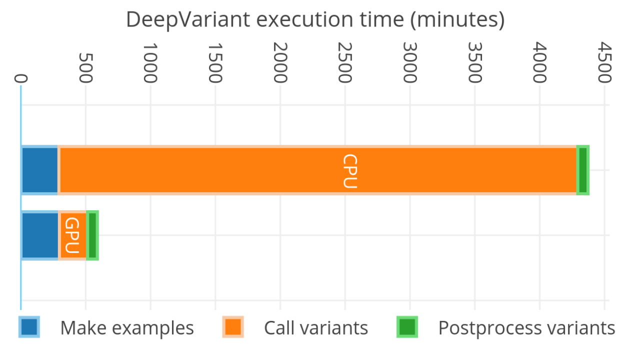

Figure 8. Single node CPU-only and GPU accelerated DeepVariant for ERR194003 (30x) dataset.

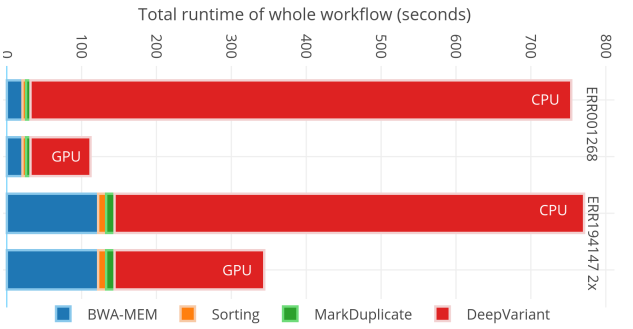

Figure 9. Total runtime for DeepVariant based complete variant calling workflow (SVCall) which uses best performance combination of nodes. For both datasets pre-processing (BWA-MEM, Sorting and MarkDuplicate) is using 16 nodes while 32 nodes are used for DeepVariant.

to the next sorting stage. The only data transformation that happens between the alignment and sorting stages is the conversion of RDDs containing Arrow RecordBatch objects to PySpark Dataframes. This transformation is handled through the Apache Arrow APIs internally. A similar key-value pairs transformation of sorted Dataframes to SAM values occurs before the MarkDuplicate stage. Compared to SparkGA2 and ADAM pre-processing results, more than 2x speedup is achieved for all cluster sizes and for both ERR001268, and ERR194003 (2x) datasets for SparkGA2 while 2-4x speedup is achieved as compared to ADAM workflow pre-processing, as shown Figure 6 and Figure 7, respectively.

Variant calling (DeepVariant)

DeepVariant is about 3x to 4x slower than GATK's HaplotypeCaller on CPU-only machines [47]. To make it scalable for clusters, we run each chromosome region independently on a different Spark worker node. In our pre-processing stage, we already store the load-balanced BAMs as individual chromosome regions. This approach provides a very fruitful base for a subsequent variant calling stage (DeepVariant in our case). For DeepVariant CPU-only version, we used a CPU cluster with different number of nodes (2, 4, 8, 16, and 32) and with multiple datasets like ERR001268, ERR194003 (2x), ERR194003 (30x) and NA12878 (300x). In Figure 10, the results show an increasing speedup for DeepVariant scalability on a Spark cluster. In DeepVariant some smaller datasets perform best with just 16 nodes, while the processing trend of other datasets show even more scalability when we increase the nodes from 16 to

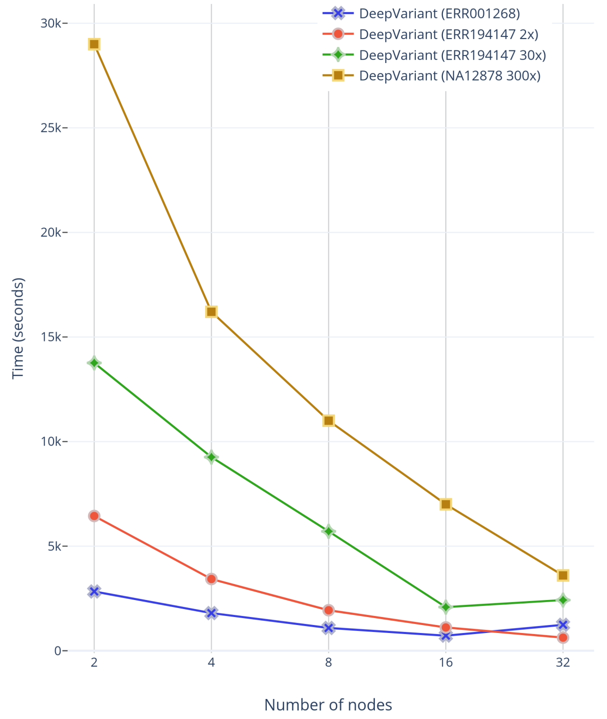

Figure 10. SVCall-DeepVariant scalability for different datasets and the number of nodes used in each run.

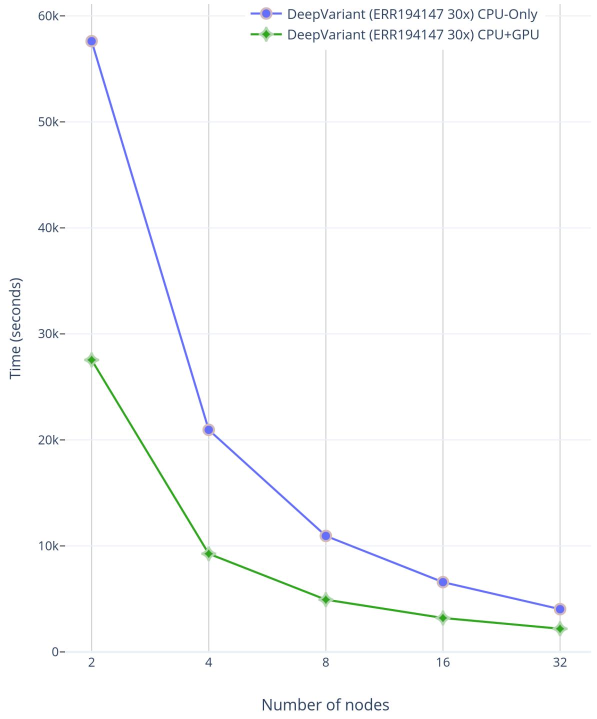

Figure 11. GPUs accelerated SVCall-DeepVariant scalability for ERR194003 (30x) dataset.

32. The total runtime is decreased up to 8x as compared to a single CPU machine. DeepVariant consist of three steps: 1) make_examples, 2) call_variants and 3) postprocess_variants. The first two steps are the most time consuming (see Figure 2). To improve their performance, the make_examples step is multi-threaded for reading inputs and creating examples, while call_variants has been accelerated for GPUs. As shown in Figure 8, we have observed in some datasets like ERR194003 (30x) that the call_variants step takes up to 95% of the total time of DeepVariant. This step can be accelerated on GPUs with almost 10x as shown in the GPU accelerated results of Figure 8. Such acceleration makes DeepVariant more feasible to be adopted in practice. We also use a GPU cluster to test our approach for DeepVariant scalability as well as acceleration. Results in Figure 11 show more than 2x speedup with GPU accelerated DeepVariant for the ERR194003 (30x) dataset as compared to CPU-only.

Variant calling workflow

The total runtime results for whole variant calling workflow using BWA-MEM, Sorting, MarkDuplicate and DeepVariant are shown in the Figure 9. Here we show the best possible nodes configuration for both, pre-processing and variant calling stages. For the dataset ERR194003 (2x), in pre-processing 16 nodes are the best fit while 32 nodes give better scalability in variant calling. Similarly for dataset ERR001268, 16 nodes provides best performance and scalability. The total runtime is decreased by up to 5x as compared to a single CPU machine.

Standalone BWA-MEM & DeepVariant

Our workflow can also be used as two independent components: a standalone BWA-MEM and a standalone DeepVariant component. The BWA-MEM component represents the fastest standalone Spark-based scalable implementation compared to other state-of-the-art BWA-MEM cluster solutions. In this solution we achieve almost linear speedups with increasing the number of nodes. The output is saved into separate SAM files which can be merged through Samtools to output a single SAM file.

In this solution, an already created BAM file can be used with DeepVariant for variant calling on cluster. As discussed earlier in Section "DeepVariant", we used Samtools to split the BAM file into our pre-defined chromosome regions to generate load-balanced chromosome regions parts. In this way we ran DeepVariant instances on Spark worker nodes. The output speedup and scalability results are the same as mentioned in Section "Variant calling (DeepVariant)".

Discussion

Here we discuss some of the advantages and limitations of our approach, in addition to the advantages of using Apache Arrow as a common in-memory data format for variant calling workflows.

Portability of the implementation

The workflow implementations discussed in this paper are portable to many HPC cluster environments. We use standard cluster solutions such as the Singularity container, and the Slurm Workload Manager to deploy and reproduce them with ease on other cluster environments.

Parallelization and scalability

Due to dividing chromosomes based on regions for load-balancing in the alignment stage, better parallelization is achieved per node in both pre-processing and variant calling stages. In the examples in this paper, we created a total of 65 such regions, which allows us to scale up to 32 nodes for the pre-processing and DeepVariant stages. When using 32 nodes, two regions are being mapped to each worker node. The total runtime of the workflow is determined by the slowest node in the cluster. As the size of the input data increases, making smaller regions can give more scalability for higher number of nodes.

Two points are important to understand the scalability and performance predictability of such applications when using the Apache Spark framework. 1. Spark always takes some fraction of time to initialize the underlying processes on its worker nodes and also spends a similar amount of time in scheduling and collecting the result. Therefore, increasing the number of nodes Spark uses, also increases this overhead time. If increasing the number of nodes results in a small overall processing time then it reaches a point where the above mentioned overhead time surpasses the processing time. 2. Data size also influences the scalability and performance of these applications and this is directly linked to our previous point. When we increase the number of nodes, the data size is always divided by the number of nodes being used. So we have to figure out the best possible scenario of performance on the cluster when choosing the number of nodes and data size being used.

System resources utilization

Existing Spark based variant calling workflows like ADAM, SparkGA2 and Halvade launch multiple instances of BWA-MEM on each Spark worker nodes which degrades the actual performance of BWA-MEM instances on each individual node. These workflows store the output of each stage to the disk which incurs sometimes I/O wait overheads as well as reading and writing to I/Os for each stage and parsing text SAM or compressed BAM also involves some additional overheads as shown in Figure 12. The figure uses the ERR194003 (2x) dataset with 16 nodes cluster (the best scalable and optimized use case for both SparkGA2 and in our approach). For comparison, we also show the system resource utilization for our approach in Figure 13. In both approaches, the first 50 seconds are spent to load the FASTA index and to read the first FASTQ data chunk. In SparkGA2, the I/O wait time is a bit higher than ours because it loads multiple indices for multiple BWA-MEM instances on each node while we just load one FASTA index on each node. After loading the files, the actual alignment process starts. The figures show that in SparkGA2 a maximum of 78% CPU resources are being used for BWA-MEM while in our approach almost 95% on average CPU resources are being used for BWA-MEM. Similarly, in Sorting only about 10% and in MarkDuplicate 50% on average CPU resources are being used in SparkGA2. In our approach, timing graph shows almost both stages take half of the total time with an average of 60-65% utilization. Because Spark uses lazy evaluations of Dataframes operations, we cannot distinguish exactly the timing for each operation separately. Due to some internal shuffling and the PySpark to Pandas Dataframes conversion via Apache Arrow, a slightly bigger amount of system time is being spent there.

Memory consumption

We use Plasma Object Store to place temporary BWA-MEM output data in-memory on each node. These objects are removed

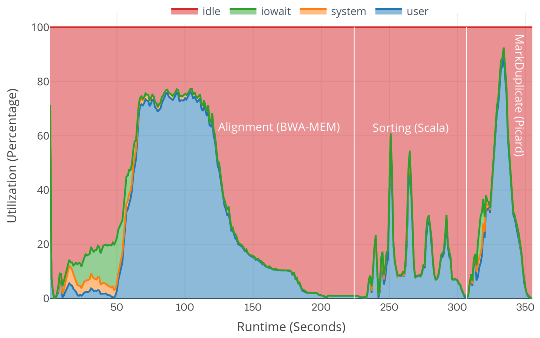

Figure 12. SparkGA2 cluster wide system resources utilization graph for pre-processing stages.

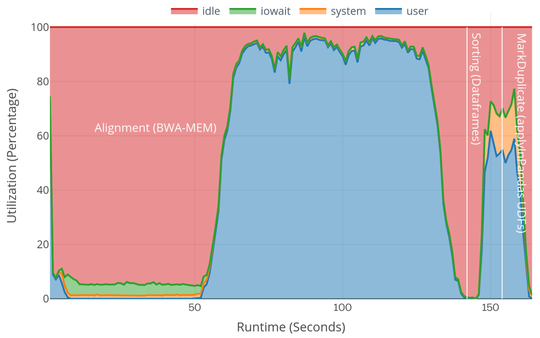

Figure 13. SVCall cluster wide system resources utilization graph for pre-processing stages.

when the Spark Dataframes creation is accomplished. During this intermediate step we use a memory space that is 2x the size of the SAM file. Similarly, during the sorting process, Spark does a lot of internal shuffling which requires additional memory. In MarkDuplicate, we use Pandas UDFs which internally use the Arrow data format for PySpark Dataframes to Pandas Dataframes conversion and vice versa. This step is also memory intensive. This workflow in pre-processing stages, requires 2x memory size as compared to SAM data produced by BWA-MEM stage on that worker node while the master node requires memory size equal to total size of the SAM data produced by all worker nodes. For DeepVariant stage, it only requires a couple of GBytes memory on both worker and master nodes.

Conclusion

A scalable and high performance DeepVariant based variant calling workflow for cluster scaled environment is presented in this paper. We employ FASTQ data streaming technique to feed data to an alignment stage followed by an in-memory data load-balancing method to store alignment output. Sorting and mark duplicate stages are implemented in such a way to get benefits from the Apache Arrow data format. The load-balanced BAM files output of the pre-processing stages is used in DeepVariant, making variant calling more efficient on a compute cluster.

Scalability analysis of our approach shows significant reduction in runtime compared to a single node. For pre-processing stages, ERR001268 and ERR194003 (2x) datasets provide up to 7x and 8x for 16 nodes, respectively. For DeepVariant, ERR001268 gives 5x, ERR194003 (2x) gives nearly 8x, ERR194003 (30x) and NA12878 (300x) gives 12x speedup for

32 nodes as compared to single node runtime. Similarly, our approach is faster than state-of-the-art workflows, such as SparkGA2, resulting in 1.8x and 2x speedup for ERR001268 and ERR194003 (2x) for pre-processing stages on 16 nodes, respectively. Our architectural approaches also increase efficient system resource utilization. For pre-processing stages, we achieve 20% to 25% better processor utilization which in turn helps to speedup overall processing.

Availability of source code and requirements

- Project name: SVCall (Scalable Variant Calling)
- Project home page: https://github.com/abs-tudelft/SVCall
- Operating system(s): Platform independent
- Programming language: Bash, Python, C, C++
- Other requirements: Singularity, Apache Spark 3.0.1, Apache Arrow 1.0.0
- License: Apache 2.0

Availability of supporting data and materials

Human Reference Genome, Build 37 is available at (GRCh37/hg19) [43]. Illumina HiSeq generated WGS paired-end read data of NA12878 with sample ERR001268 [40], Illumina HiSeq 2000 paired-end NA12878 with sample ERR194003 [41] with sequencing coverage of 30x and Illumina HiSeq 300x HG002 sample of NA12878 [42] are used to evaluate this work and are publicly available.

Declarations

List of abbreviations

SVCall: Scalable Variant Calling; NGS: Next Generation Sequencing; MPI: Message Passing Interface; I/O: Input/Output; JVM: Java Virtual Machine; HPC: High Performance Computing; SAM: Sequence Alignment/Map; BAM: Binary Alignment/Map; VCF: Variant Calling File; NFS: Network File System; DNA: Deoxyribonucleic acid; HDFS: Hadoop Distributed File System; RDD: re-silient distributed datasets; PCR: olymeraseChain Reaction; ONT: Oxford Nanopore; SNP: single nucleotide polymorphisms;, indels: insertions and deletions; SV: structural variants; CNV: Copy Number Variations; UPC: Unified Parallel C; UDF: user-defined functions; DF: Dataframe; RB: Record-Batches; IPC: inter-process communication;

Ethical Approval

We use all publicly available and authorized human genome datasets.

Consent for publication

"Not applicable"

Competing Interests

'The author(s) declare that they have no competing interests'.

Funding

The PhD research of Tanveer Ahmad is generously funded by Punjab Educational Endowment Fund (PEEF), Pakistan.

Author's Contributions

Z.A.A and P.H. conceived and supervised this work. T.A. designed and developed whole variant calling workflow. All authors read and approved the final manuscript.

Acknowledgements

This work was carried out on the Dutch national e-infrastructure with the support of SURF Cooperative. Thanks to Hamid Mushtaq from Maastricht University Medical Center+ (Maastricht UMC+), Netherlands for helping in running the SparkGA2 on HPC cluster.

References

1. Gropp W, Lusk E. Fault Tolerance in Message Passing Interface Programs. The International Journal of High Performance Computing Applications 2004;18(3):363–372. https://doi.org/10.1177/1094342004046045.
2. Cappello F, Al G, Gropp W, Kale S, Kramer B, Snir M. Toward Exascale Resilience: 2014 Update. Supercomput Front Innov: Int J 2014 Apr;1(1):5–28. https://doi.org/10.14529/jsfi140101.
3. Apache, Apache Hadoop [Accessed: 2nd April 2019]; 2019. https://hadoop.apache.org/.
4. Decap D, Reumers J, Herzeel C, Costanza P, Fostier J. Halvade: scalable sequence analysis with MapReduce. Bioinformatics (Oxford, England) 2015 Aug;31(15):2482–2488. https://pubmed.ncbi.nlm.nih.gov/25819078, 25819078[pmid].
5. Apache, Apache Spark: Lightning-fast unified analytics engine [Accessed: 2nd April 2019]; 2019. https://spark.apache.org/.
6. Mushtaq H, Liu F, Costa C, Liu G, Hofstee P, Al-Ars Z. SparkGA: A Spark Framework for Cost Effective, Fast and Accurate DNA Analysis at Scale. In: Proceedings of the 8th ACM International Conference on Bioinformatics, Computational Biology,and Health Informatics ACM-BCB '17, New York, NY, USA: ACM; 2017. p. 148–157. http://doi.acm.org/10.1145/3107411.3107438.
7. Massie M, Nothaft F, Hartl C, Kozanitis C, Schumacher A, Joseph AD, et al. ADAM: Genomics Formats and Processing Patterns for Cloud Scale Computing. UCB/EECS-2013-207, EECS Department, University of California, Berkeley; 2013.
8. Abuín JM, Pichel JC, Pena TF, Amigo J. SparkBWA: Speeding Up the Alignment of High-Throughput DNA Sequencing Data. PLOS ONE 2016 05;11(5):1–21. https://doi.org/10.1371/journal.pone.0155461.
9. Institute B, BWA on Spark; 2018. https://gatk.broadinstitute.org/hc/en-us/articles/360037225092-BwaSpark-BETA-.
10. Zhang L, Liu C, Dong S. PipeMEM: A Framework to Speed Up BWA-MEM in Spark with Low Overhead. Genes 2019 Nov;10(11):886. http://dx.doi.org/10.3390/genes10110886.
11. Langmead B, Salzberg SL. Fast gapped-read alignment with Bowtie 2. Nat Methods 2012 Mar;9(4):357–359. https://www.ncbi.nlm.nih.gov/pubmed/22388286, 22388286[pmid].
12. Li H, Durbin R. Fast and accurate short read alignment with Burrows-Wheeler transform. Bioinformatics 2009 05;25(14):1754–1760. https://doi.org/10.1093/bioinformatics/btp324.
13. Myers G. Efficient Local Alignment Discovery amongst Noisy Long Reads. In: Brown D, Morgenstern B, editors. Algorithms in Bioinformatics Berlin, Heidelberg: Springer

Berlin Heidelberg; 2014. p. 52–67.
14. Li H. The Sequence Alignment/Map format and SAMtools. Bioinformatics 2009 01;25:2078 – 2079.
15. Picard toolkit. Broad Institute; Accessed: April 11, 2019. http://broadinstitute.github.io/picard/.
16. Tarasov A, Vilella AJ, Cuppen E, Nijman IJ, Prins P. Sambamba: fast processing of NGS alignment formats. Bioinformatics 2015 Jun;31(12):2032–2034. https://www.ncbi.nlm.nih.gov/pubmed/25697820, 25697820[pmid].
17. Faust GG, Hall IM. SAMBLASTER: fast duplicate marking and structural variant read extraction. Bioinformatics 2014 Sep;30(17):2503–2505. 24812344[pmid].
18. Koboldt DC, Zhang Q, Larson DE, Shen D, McLellan MD, Lin L, et al. VarScan 2: Somatic mutation and copy number alteration discovery in cancer by exome sequencing. Genome Research 2012 Feb;22(3):568–576. https://doi.org/10.1101/gr.129684.111.
19. Lai Z, Markovets A, Ahdesmaki M, Chapman B, Hofmann O, McEwen R, et al. VarDict: a novel and versatile variant caller for next-generation sequencing in cancer research. Nucleic Acids Research 2016 Apr;44(11):e108–e108. https://doi.org/10.1093/nar/gkw227.
20. Cibulskis K, Lawrence MS, Carter SL, Sivachenko A, Jaffe D, Sougnez C, et al. Sensitive detection of somatic point mutations in impure and heterogeneous cancer samples. Nature Biotechnology 2013 Feb;31:213 EP –. https://doi.org/10.1038/nbt.2514.
21. Sahraeian SME, Liu R, Lau B, Podesta K, Mohiyuddin M, Lam HYK. Deep convolutional neural networks for accurate somatic mutation detection. Nature Communications 2019 Mar;10(1):1041. https://doi.org/10.1038/s41467-019-09027-x.
22. Sahraeian SME, Fang LT, Mohiyuddin M, Hong H, Xiao W. Robust Cancer Mutation Detection with Deep Learning Models Derived from Tumor-Normal Sequencing Data. bioRxiv 2019;https://www.biorxiv.org/content/early/2019/06/11/667261.1.
23. Garrison E, Marth G, Haplotype-based variant detection from short-read sequencing; 2012.
24. Kim S, Scheffler K, Halpern AL, Bekritsky MA, Noh E, Källberg M, et al. Strelka2: fast and accurate calling of germline and somatic variants. Nature Methods 2018;15(8):591–594. https://doi.org/10.1038/s41592-018-0051-x.
25. Poplin R, Chang PC, Alexander D, Schwartz S, Colthurst T, Ku A, et al. A universal SNP and small-indel variant caller using deep neural networks. Nature Biotechnology 2018 Sep;36:983 EP –. https://doi.org/10.1038/nbt.4235.
26. Wei Z, Wang W, Hu P, Lyon GJ, Hakonarson H. SNVer: a statistical tool for variant calling in analysis of pooled or individual next-generation sequencing data. Nucleic Acids Research 2011 Aug;39(19):e132–e132. https://doi.org/10.1093/nar/gkr599.
27. Wilm A, Aw PPK, Bertrand D, Yeo GHT, Ong SH, Wong CH, et al. LoFreq: a sequence-quality aware, ultra-sensitive variant caller for uncovering cell-population heterogeneity from high-throughput sequencing datasets. Nucleic Acids Research 2012 Oct;40(22):11189–11201. https://doi.org/10.1093/nar/gks918.
28. FDA, PrecisionFDA Truth Challenge; 2019. https://precision.fda.gov/challenges/truth.
29. FDA, PrecisionFDA Truth Challenge V2: Calling Variants from Short and Long Reads in Difficult-to-Map Regions; 2019. https://precision.fda.gov/challenges/10.
30. X L, Qiu K, Liang P, D P. Speeding up large-scale next generation sequencing data analysis with pBWA. Journal of biocomputing 2012 01;1.
31. Darling A, Carey L, Feng W. The Design, Implementation, and Evaluation of mpiBLAST. Proc Cluster World 2003 12;2003.
32. Liu Y, Popp B, Schmidt B. CUSHAW3: Sensitive and Accurate Base-Space and Color-Space Short-Read Alignment with Hybrid Seeding. PLOS ONE 2014 01;9(1):1–9. https://doi.org/10.1371/journal.pone.0086869.
33. Apache, Apache Arrow: A cross-language development platform for in-memory data [Accessed 29th Dec. 2019]; 2019. https://arrow.apache.org/.
34. Apache, PySpark Usage Guide for Pandas with Apache Arrow [Accessed: 2nd April 2019]; 2019. https://spark.apache.org/docs/latest/sql-pyspark-pandas-with-arrow.html.
35. Jin L, Introducing Pandas UDF for PySpark; 2018. https://bit.ly/3930obR.
36. Shen W, Le S, Li Y, Hu F. SeqKit: A Cross-Platform and Ultrafast Toolkit for FASTA/Q File Manipulation. PLOS ONE 2016 10;11(10):1–10. https://doi.org/10.1371/journal.pone.0163962.
37. Apache, Plasma In-Memory Object Store [Accessed 29th Dec. 2019]; 2019. https://arrow.apache.org/blog/2017/08/08/plasma-in-memory-object-store/.
38. FDA, precisionFDA: A community platform for NGS assay evaluation and regulatory science exploration; 2019. https://precision.fda.gov/.
39. UCSC, faSplit; 2018. http://hgdownload.cse.ucsc.edu/admin/exe/linux.x86_64/.
40. Illumina, Illumina Cambridge Ltd. [Accessed 24th May 2019]; 2012. ftp://ftp.1000genomes.ebi.ac.uk/vol1/ftp/phase3/data/NA12878/sequence_read/.
41. (ENA) TENA, Illumina 30X; 2020. https://www.ebi.ac.uk/ena/browser/view/ERR194147.
42. GIAB, NHGRI Illumina 300X BAM; 2020. ftp://ftp-trace.ncbi.nlm.nih.gov/giab/ftp/data/NA12878/NIST_NA12878_HG001_HiSeq_300x/NHGRI_Illumina300X_novoalign_bams/.
43. UCSC, UCSC hg19 (GRCh37); 2020. https://hgdownload.soe.ucsc.edu/goldenPath/hg19/bigZips/.
44. SurfSara, Cartesius: the Dutch supercomputer; 2020. https://userinfo.surfsara.nl/systems/cartesius.
45. Lustre, Lustre parallel filesystem; 2020. https://www.lustre.org/.
46. Slurm, Slurm workload manager; 2020. https://www.schedmd.com/.
47. CARROLL A, THANGARAJ N, Evaluating DeepVariant: A New Deep Learning Variant Caller from the Google Brain Team; 2017. https://bit.ly/3n4XtDT.
