# Supplementary material for: VC@Scale: Scalable and high-performance variant calling on cluster environments
Source: Gigascience. 2021 Sep 7;10(9):giab057. doi: 10.1093/gigascience/giab057 (PMC8424057; doi:10.1093/gigascience/giab057)
Supplement: giab057_Response_to_Reviewer_Comments_Revision_1 [file giab057_response_to_reviewer_comments_revision_1.pdf]

Author's Response To Reviewer Comments

Close

Dear Hans Zauner,

Thanks for the response.

We have updated our manuscript as per reviewer # 2 corrections/suggestions. Please check our reply below:

Reviewer #2:

a) Some figures (e.g. Fig. 6 and Fig. 7) still refer to "SVCall" rather than VC@Scale. Please carefully revise. Thanks, we have corrected and revised the manuscript.
b) There seems to be no comparison to single node runtime: all plots start from 2 nodes. As such, we can not compare to the runtime of the regular pipelines (which are also not provided). Figure 1 shows the total run-time with different datasets on a single node with regular pipeline while Fighure 8 and 9 show the single node vs single-node with GPU total run-times for regular pipelines.
c) In Fig. 2, it is argued that part of the reason to use Apache Arrow is to avoid the (de)serialization of data from the JVM to pySpark. But Spark Java/Scala also uses the JVM so the data does not need to be (de)serialized when using those programming languages. However, Apache Arrow is still very useful with its in-memory columnar format that can be access through different APIs. Maybe this information can be included in the Figure caption. Thanks for the suggestion. Figure caption has been updated.
d) In the response letter, it is indicated that using 128 partitions instead of 65 improves the load balance of partitions and even gives better performance. Is there an optimum, did you test this, how should users determine this? We have tested this internally, We didn't included as it will also require more updates in the original scripts. We will update our github repo in future releases of code with some other major updates we already plan.

The updated version of the manuscript has been uploaded in giga's editorialmanager portal. Thanks.

Regards,
--
Tanveer Ahmad
PhD Student
Department of Quantum & Computer Engineering
EEMCS, TU Delft, The Netherlands

Close
