# Supplementary material for: VC@Scale: Scalable and high-performance variant calling on cluster environments
Source: Gigascience. 2021 Sep 7;10(9):giab057. doi: 10.1093/gigascience/giab057 (PMC8424057; doi:10.1093/gigascience/giab057)
Supplement: giab057_Response_to_Reviewer_Comments_Revision_2 [file giab057_response_to_reviewer_comments_revision_2.pdf]

Author's Response To Reviewer Comments

Close

Dear Editor,

All required changes has been done. ORCID have already been provided. I can not find a place on title page to add ORCID ids. Thanks.

Regards,
Tanveeer Ahmad

Close
