# Supplementary material for: VC@Scale: Scalable and high-performance variant calling on cluster environments
Source: Gigascience. 2021 Sep 7;10(9):giab057. doi: 10.1093/gigascience/giab057 (PMC8424057; doi:10.1093/gigascience/giab057)
Supplement: giab057_Reviewer_1_Report_Original_Submission — Medhat Mahmoud -- 2/21/2021 Reviewed [file giab057_reviewer_1_report_original_submission.pdf]

Reviewer Report

Title: VC@Scale: Scalable and High Performance Variant Calling on Cluster Environments

Version: Original Submission **Date:** 2/21/2021

Reviewer name: Medhat Mahmoud

Reviewer Comments to Author:

SVCall: Scalable DeepVariant based Variant Calling on Spark cluster using Apache Arrow
Variant calling is an essential step in genome analysis, which is associated with disease discovery and evolution. Various tools have been developed to identify variations. With the advance in machine and deep learning, new methods have been suggested, which depend on these approaches, the caveat is the speed, these tools take longer running time compared with other conventional methods. Ahmed et al. are using Apache spark to distribute DeepVariant running to reduce running time.
The name is misleading SVcall could stand for Structural Variant call, the tools actually works on small variations.
The introduction section identified well the MPI, MapReduce, and Pandas technology and its integration with Spark, while not much about the variant calling.
While the paper is focusing on NGS, the authors mentioned aligners for long-reads and which is not relevant to main topic and did not add to the paper
The authors mentioned three steps as the main/important thing for variant calling:
" 1. Alignment of raw FASTQ data against a reference genome,
2. chromosome based coordinate sorting, and 3. Polymerase Chain Reaction (PCR) duplicates removal. These steps are important in every variant calling workflow." actually the third point is not that important;
Remove PCR duplications is not needed anymore:
https://bmcbioinformatics.biomedcentral.com/articles/10.1186/s12859-016-1097-3
Also, it is not mandatory by DeepVaraint
https://github.com/google/deepvariant/issues/35
The SVcall is depending mainly on two tools, one of them (the aligner is changing to BWA-mem2 reducing memory usage 4 times already, which SVcall try to reduce) and I quote "Bwa-mem2 is the next version of the bwa-mem algorithm in bwa. It produces alignment identical to bwa and is ~1.3-3.1x faster depending on the use-case, dataset and the running machine."
Vasimuddin Md, Sanchit Misra, Heng Li, Srinivas Aluru. Efficient Architecture-Aware Acceleration of BWA-MEM for Multicore Systems. IEEE Parallel and Distributed Processing Symposium (IPDPS), 2019.
The approach introduced by the authors can not be applied to other variant callers.
The authors did not point to the novelty of work, except increasing running time 2x, but SVcall did not change result accuracy or add much to end results.

Level of Interest

Please indicate how interesting you found the manuscript: Choose an item.

Quality of Written English

Please indicate the quality of language in the manuscript: Choose an item.

Declaration of Competing Interests

Please complete a declaration of competing interests, considering the following questions:

- Have you in the past five years received reimbursements, fees, funding, or salary from an organisation that may in any way gain or lose financially from the publication of this manuscript, either now or in the future?
- Do you hold any stocks or shares in an organisation that may in any way gain or lose financially from the publication of this manuscript, either now or in the future?
- Do you hold or are you currently applying for any patents relating to the content of the manuscript?
- Have you received reimbursements, fees, funding, or salary from an organization that holds or has applied for patents relating to the content of the manuscript?
- Do you have any other financial competing interests?
- Do you have any non-financial competing interests in relation to this paper?

If you can answer no to all of the above, write 'I declare that I have no competing interests' below. If your reply is yes to any, please give details below.

I declare that I have no competing interests

I agree to the open peer review policy of the journal. I understand that my name will be included on my report to the authors and, if the manuscript is accepted for publication, my named report including any attachments I upload will be posted on the website along with the authors' responses. I agree for my report to be made available under an Open Access Creative Commons CC-BY license (http://creativecommons.org/licenses/by/4.0/). I understand that any comments which I do not wish to be included in my named report can be included as confidential comments to the editors, which will not be published.

Choose an item.

To further support our reviewers, we have joined with Publons, where you can gain additional credit to further highlight your hard work (see: https://publons.com/journal/530/gigascience). On publication of this paper, your review will be automatically added to Publons, you can then choose whether or not to claim your Publons credit. I understand this statement.

Yes Choose an item.
