# Supplementary material for: VC@Scale: Scalable and high-performance variant calling on cluster environments
Source: Gigascience. 2021 Sep 7;10(9):giab057. doi: 10.1093/gigascience/giab057 (PMC8424057; doi:10.1093/gigascience/giab057)
Supplement: giab057_Reviewer_2_Report_Original_Submission — Jan Fostier -- 2/23/2021 Reviewed [file giab057_reviewer_2_report_original_submission.pdf]

Reviewer Report

Title: VC@Scale: Scalable and High Performance Variant Calling on Cluster Environments

Version: Original Submission **Date:** 2/23/2021

Reviewer name: Jan Fostier

Reviewer Comments to Author:

The paper does not clearly mention whether somatic variant calling is supported or not. It is stated that DeepVariant can call both germline and somatic variants but it is unclear whether SVCall supports this as well. The samples seem to indicate only germline variant calling is supported.
Static load balancing using 65 parts has certain limitations as described in the summary. This parameter seems to be chosen by the fact that at most 32 nodes are used, approximately 2x the number of nodes. Would it make sense to lower the number of partitions for fewer nodes, or increase them for more nodes (i.e., variable partitioning of the genome)? For smaller datasets, it appears that scaling to a larger number of nodes (e.g. 32) becomes difficult; is this a consequence of poor load balancing? As indicated in the summary, a balance between the optimal number of partitions and having not too much overhead should be attained. SparkGA2 splits the regions based on the number of reads rather than genome size, which is a more accurate representation of the CPU time required to process that region. Why is this strategy abandoned in SVCall?
It is argued that running multiple instances of BWA degrades the performance compared to using multiple threads within a single BWA instance. In the summary it is stated that the I/O waiting time is higher in SparkGA (where multiple instances are used) than it is in the SVCall implementation. This sounds odd as in both cases it takes about 50s to load the reference genome and the rest of the CPU cores are idle anyway. Thus, the I/O wait time does not seem to matter at that point.
Some quick tests show that 2 instances running 2 threads run in the same time as 1 instance using 4 threads on our systems. The observation that running multiple instances yields lower performance than multithreading might in fact be caused by poor load balancing. Either more evidence is needed to support the claim that multithreading is better than multiple instances or the paragraph should be rephrased. However, it is clear that the sorting step and the "mark duplicates" steps seem to have a higher performance.

Level of Interest

Please indicate how interesting you found the manuscript: Choose an item.

Quality of Written English

Please indicate the quality of language in the manuscript: Choose an item.

Declaration of Competing Interests

Please complete a declaration of competing interests, considering the following questions:

- Have you in the past five years received reimbursements, fees, funding, or salary from an organisation that may in any way gain or lose financially from the publication of this manuscript, either now or in the future?
- Do you hold any stocks or shares in an organisation that may in any way gain or lose financially from the publication of this manuscript, either now or in the future?
- Do you hold or are you currently applying for any patents relating to the content of the manuscript?
- Have you received reimbursements, fees, funding, or salary from an organization that holds or has applied for patents relating to the content of the manuscript?
- Do you have any other financial competing interests?
- Do you have any non-financial competing interests in relation to this paper?

If you can answer no to all of the above, write 'I declare that I have no competing interests' below. If your reply is yes to any, please give details below.

I declare that I have no competing interests

I agree to the open peer review policy of the journal. I understand that my name will be included on my report to the authors and, if the manuscript is accepted for publication, my named report including any attachments I upload will be posted on the website along with the authors' responses. I agree for my report to be made available under an Open Access Creative Commons CC-BY license (http://creativecommons.org/licenses/by/4.0/). I understand that any comments which I do not wish to be included in my named report can be included as confidential comments to the editors, which will not be published.

Choose an item.

To further support our reviewers, we have joined with Publons, where you can gain additional credit to further highlight your hard work (see: https://publons.com/journal/530/gigascience). On publication of this paper, your review will be automatically added to Publons, you can then choose whether or not to claim your Publons credit. I understand this statement.

Yes Choose an item.
