# Supplementary material for: VC@Scale: Scalable and high-performance variant calling on cluster environments
Source: Gigascience. 2021 Sep 7;10(9):giab057. doi: 10.1093/gigascience/giab057 (PMC8424057; doi:10.1093/gigascience/giab057)
Supplement: giab057_Reviewer_3_Report_Original_Submission — Ted Ahn -- 3/5/2021 Reviewed [file giab057_reviewer_3_report_original_submission.pdf]

Reviewer Report

Title: VC@Scale: Scalable and High Performance Variant Calling on Cluster Environments

Version: Original Submission **Date:** 3/5/2021

Reviewer name: Ted Ahn

Reviewer Comments to Author:

This paper presents an implementation of next-generation sequencing (NGS) data pre-processing and variant calling workflows. Especially, the authors used Apache Arrow, in-memory standard columnar data format, in Apache Spark with selected tools (BWA for mapping and DeepVariant for variant calling) for scalable variant calling workflow.
Background is well described. Methods section explains the detailed steps with figures clearly. Results show that the proposed SVCall framework outperforms (especially, wall-clock time based) other SparkGA2 and ADAM frameworks. Benchmarking results are well presented with figures. The review especially like the Discussion section that authors considered the scalability and performance prediction and the limits based on Spark's own initialization step and more.
Overall, I think the paper is suitable for publication and well-written. The manuscript is clear and quite readable, though some improvements could be made as below.
General concerns:
1) The paper would benefit if the authors consider how to prove the "correctness" of output from the proposed SVCall framework compared to the original pipeline without applying any parallelization, though allowing multi-threads if tools support them. One possible way is to show identical VCF results and summary from original pipeline and from the SVCall. The authors do not need to show the comparison of performance and accuracy among different pipelines (e.g., BWA-Stelka2 vs BWA-DeepVariant). The current pick (BWA-DeepVariant) seems to be a good choice to me. If the authors just prove that researchers can achieve identical results with such a speed-up using this proposed framework, those researchers (pathologists/biologists) will be convinced to use the tool.
2) The order of the figures are not following the order of figures that are cited in the manuscript. The order of figures should follow the order in which they appear in the manuscript. I am not sure whether GigaScience journal allows it or not, but it would be good to be updated.
Minor comments:
* p.3: "Deep-Variant does not required some additional pre-processing steps" should be updated as "Deep-Variant does not require some additional pre-processing steps".
* p.3 and p4: Figure 2 has been mentioned in page 3 before the Figure 1 is cited in page 4. The order of figures should follow the order in which they appear in the manuscript.
* p.4: "These functions shares the same…" should be updated as "These functions share the same…".
* p.5: Figure 5 is also cited before of Figure 4.
* p.5: "in the options parameters"? Do you mean "in the options and parameters" or something else?
* p.7: "threads.Therefore" should have a space.

Level of Interest

Please indicate how interesting you found the manuscript: Choose an item.

Quality of Written English

Please indicate the quality of language in the manuscript: Choose an item.

Declaration of Competing Interests

Please complete a declaration of competing interests, considering the following questions:

- Have you in the past five years received reimbursements, fees, funding, or salary from an organisation that may in any way gain or lose financially from the publication of this manuscript, either now or in the future?
- Do you hold any stocks or shares in an organisation that may in any way gain or lose financially from the publication of this manuscript, either now or in the future?
- Do you hold or are you currently applying for any patents relating to the content of the manuscript?
- Have you received reimbursements, fees, funding, or salary from an organization that holds or has applied for patents relating to the content of the manuscript?
- Do you have any other financial competing interests?
- Do you have any non-financial competing interests in relation to this paper?

If you can answer no to all of the above, write 'I declare that I have no competing interests' below. If your reply is yes to any, please give details below.

I declare that I have no competing interests.

I agree to the open peer review policy of the journal. I understand that my name will be included on my report to the authors and, if the manuscript is accepted for publication, my named report including any attachments I upload will be posted on the website along with the authors' responses. I agree for my report to be made available under an Open Access Creative Commons CC-BY license (http://creativecommons.org/licenses/by/4.0/). I understand that any comments which I do not wish to be included in my named report can be included as confidential comments to the editors, which will not be published.

Choose an item.

To further support our reviewers, we have joined with Publons, where you can gain additional credit to further highlight your hard work (see: https://publons.com/journal/530/gigascience). On publication of

this paper, your review will be automatically added to Publons, you can then choose whether or not to claim your Publons credit. I understand this statement.

Yes Choose an item.
